# Expanding Cas12a Activity Control with an RNA G‐Quadruplex at the 5′ end of CRISPR RNA

**DOI:** 10.1002/advs.202411305

**Published:** 2024-12-25

**Authors:** Wenjuan Huang, Jiaqi Wang, Cheng Wang, Yuanfang Liu, Wentao Li, Qiaozhen Chen, Junqiu Zhai, Zhenyang Xiang, Chaoxing Liu

**Affiliations:** ^1^ Taizhou Hospital of Zhejiang Province Affiliated to Wenzhou Medical University Linhai 317000 P. R. China; ^2^ Guangdong Provincial Key Laboratory of Digestive Cancer Research Digestive Diseases Center Scientific Research Center The Seventh Affiliated Hospital of Sun Yat‐sen University Shenzhen Guangdong 518107 P. R. China; ^3^ Department of Clinical Laboratory The Seventh Affiliated Hospital of Sun Yat‐sen University Shenzhen Guangdong 518107 P. R. China; ^4^ School of Chemistry and Biological Engineering University of Science and Technology Beijing Beijing 100083 P. R. China; ^5^ School of Pharmaceutical Sciences Guangzhou University of Chinese Medicine Guangzhou 510006 P. R. China

**Keywords:** CRISPR RNA, CRISPR‐Cas12a, one‐pot detection, precise control, RNA G‐quadruplex

## Abstract

Precise control of Cas12a activity is essential for the improvement of the detection limit of clinical diagnostics and the minimization of errors. This study addresses the challenge of controlling Cas12a activity, especially in the context of nucleic acid detection where the inherent incompatibility between isothermal amplification and CRISPR reactions complicates accurate diagnostics. An RNA G‐quadruplex (RG4) structure at the 5′ end of crRNA is introduced to modulate Cas12a activity accurately without the need for chemical modifications. The results indicate that the presence of RG4 does not significantly impact Cas12a's cleavage activity but can be controlled by RG4 stabilizers, enabling the suppression and subsequent restoration of Cas12a activity with potential for precise activity control. Moreover, the use of RG4 is expanded by incorporating it into split crRNA, introducing RG4 directly at the 5′ end of the direct repeat (DR) region, enabling tailored activity regulation for different targets by matching with various Spacer regions. Additionally, a light‐controlled one‐pot method for activating Cas12a is developed, thereby enhancing the accuracy and sensitivity of clinical samples. This study showcases the pioneering use of RG4 in manipulating Cas12a activity, streamlining diagnostics, and paving the way for advances in clinical nucleic acid testing.

## Graphical abstract

The RNA G‐quadruplex structure at the 5' end of crRNA is used for the first time to precisely control Cas12a activity, simplifying nucleic acid detection and improving clinical sensitivity, paving the way for advanced CRISPR applications. The study highlights the potential of RNA G‐quadruplex for precise enzyme control, which holds great promise for diagnostics.

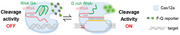

## 1 Introduction

RNA G‐quadruplex (RG4) is a prevalent non‐canonical secondary structure that is widely observed in both mRNA and non‐coding RNAs.^[^ ^1^ ^]^ It primarily forms in guanine‐rich regions and plays a significant role in translation regulation, mRNA processing, alternative splicing, protein binding, and maintaining chromosomal end integrity.^[^ ^2^, ^3^, ^4^, ^5^, ^6^ ^]^ RG4s are more stable, compact, and less hydrated than their DNA counterparts. Their formation and stability are enhanced by the presence of the ribose's 2′ hydroxyl group.^[^ ^7^, ^8^ ^]^ The stability of RG4s is less dependent on environmental conditions, which is due to their topological structure.^[^ ^9^ ^]^

Research on RG4s is crucial for understanding RNA regulation mechanisms, advancing biomedicine, and developing new drugs and treatments.^[^ ^10^, ^11^, ^12^, ^13^ ^]^ These structures can be utilized to precisely control cellular processes.^[^ ^14^, ^15^, ^16^, ^17^ ^]^ RG4s are prominent in telomeres and gene promoters in the human genome, where they regulate gene transcription, translation, replication, and genome stability.^[^ ^6^, ^18^ ^]^ They are also found in viral genomes, such as Zika and Ebola, where they impact viral lifecycle and latency.^[^ ^19^, ^20^, ^21^, ^22^ ^]^ The study of RG4 is paving the way for drug development, biomaterials, and viral therapy.^[^ ^1^ ^]^ RG4 is a unique nucleic acid tool that offers precise control over biological processes.^[^ ^9^, ^23^, ^24^ ^]^

Cas12a, also known as Cpf1, is a nuclease found in the natural bacterial immune system.^[^ ^25^ ^]^ It exhibits *cis‐*cleavage activity, enabling the recognition and cleavage of target DNA with the assistance of ≈42 nt CRISPR guide RNA (crRNA) which consists of a universal Direct Repeat (DR) Region (≈19–21 nt) and a programmable Spacer Region (18–23 nt).^[^ ^26^, ^27^, ^28^ ^]^ Cas12a is commonly used in gene editing because of its low off‐target rate and its ability to function with only crRNAs without the need for *trans‐*activating CRISPR RNA (tracrRNA).^[^ ^29^, ^30^, ^31^ ^]^ Additionally, activated Cas12a possesses *trans‐*cleavage activity which can digest non‐specific single‐stranded DNA,^[^ ^32^, ^33^ ^]^ RNA,^[^ ^34^ ^]^ λDNA,^[^ ^35^ ^]^ and 3′ overhang dsDNA,^[^ ^36^ ^]^ making it increasingly relevant in rapid nucleic acid detection for various applications, including infectious disease prevention, early cancer screening, food contamination detection, forensic examination, and therapeutic drug monitoring.^[^ ^37^, ^38^, ^39^, ^40^, ^41^, ^42^, ^43^ ^]^

Accurate control of Cas12a's activity is crucial for precise gene editing and gene therapy, minimizing off‐target effects.^[^ ^44^, ^45^ ^]^ In the field of nucleic acid detection, precise control of Cas12a's activity becomes even more imperative due to the inherent incompatibility between isothermal amplification systems and CRISPR reactions.^[^ ^38^, ^39^, ^46^ ^]^ In the initial stage of isothermal amplification, the cleavage activity of Cas12a may impede nucleic acid amplification, especially in scenarios with low input copy numbers. Initial versions of HOLMES and DETECTR required separate tubes for CRISPR reactions and amplification, thereby increasing the complexity of diagnostics and potentially generating false‐positive results caused by liquid transfer and aerosol contamination.^[^ ^32^, ^33^ ^]^ In response to these challenges, various optimization strategies have been explored, including the selection of suboptimal protospacer adjacent motifs,^[^ ^47^ ^]^ physical isolation of reagents,^[^ ^48^ ^]^ specialized buffers,^[^ ^49^ ^]^ and phase‐separation solutions.^[^ ^50^ ^]^ However, conventional methods may hinder the CRISPR reaction or present limitations in specific clinical sample testing, leaving compatibility issues unresolved. Precise control of Cas12a's activity can fundamentally resolve these concerns. Researchers introduced several chemical modifications to Cas12a's crRNA or its complementary strand. These modifications temporarily inactivated the CRISPR‐Cas12a system, which was subsequently restored through exposure to UV light after nucleic acid amplification.^[^ ^38^, ^39^, ^46^ ^]^ However, previous methods all require the introduction of chemically modified nucleic acids or pre‐labeling of crRNA with chemicals, thus complicating the process of nucleic acid testing, especially in clinical contexts. There exists a knowledge gap in the field regarding the potential for directly inserting specific secondary structures into crRNA to directly control Cas12a activity, potentially leading to new breakthroughs in clinical nucleic acid diagnostics.

Given the exceptional properties of RG4 in regulating RNA transcription and translation, as well as the ongoing clinical trials of small molecule drugs^[^ ^51^ ^]^ and antibodies^[^ ^52^ ^]^ targeting RG4,^[^ ^15^, ^53^, ^54^ ^]^ we propose the introduction of RG4 at the 5′ end of Cas12a's crRNA. RG4 represents a unique secondary structure that holds great promise for precisely regulating Cas12a activity without the need for chemical modifications of crRNA. Importantly, the fusion of crRNA and RG4 can be transcribed directly from DNA without any chemical modification. In addition, we can effectively control the conformation of RG4 using potassium ions or RG4 stabilizers to achieve precise control of Cas12a activity.

## 2 Results and Discussion

### 2.1 Effects of RG4 Present at the 5′ end of crRNA on the Activity of LbCas12a

**Figure 1:**
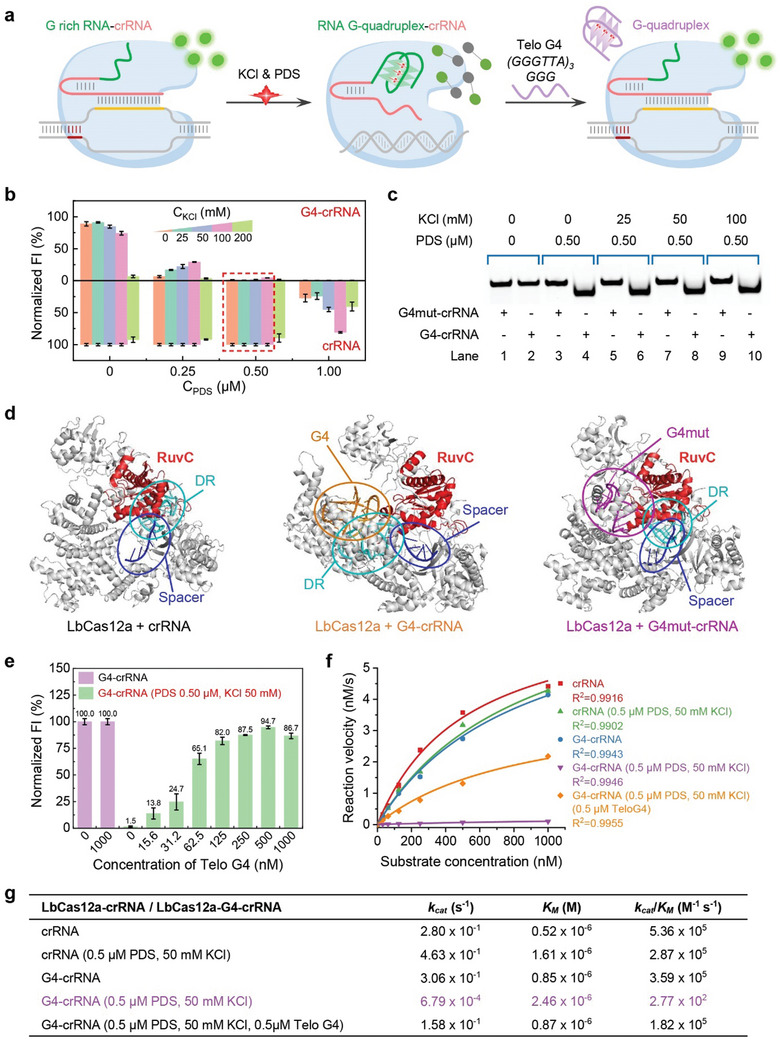
a) Schematic diagram illustrating the regulation of Cas12a activity by the 5′ end RG4 sequence of the crRNA and RG4 stabilizer. The incorporation of an RG4 sequence at the 5′ end of Cas12a crRNA does not affect the cleavage activity under normal conditions. However, the inhibition of Cas12a activity in the presence of G4 stabilizers, such as KCl and PDS, is due to the formation of a stable RG4 structure. Furthermore, excess single‐stranded telo G4 can displace the G4 stabilizer from G4‐crRNA, thereby restoring Cas12a's cleavage capability. b) Bar graph comparing fluorescence intensities of canonical crRNA (bottom) and G4‐RNA (up) under varying concentrations of KCl and PDS. Upon the formation of the crRNA‐Cas12a complex and target recognition, Cas12a's *trans‐*cleavage activity cleaves the F‐Q reporter probe, which is quenched in its native state but emits a fluorescence signal upon Cas12a activation, resulting in the separation of FAM from BHQ1. Error bars are standard deviation (SD) (*n* = 3). c) Native PAGE analysis of the secondary structure of G4‐RNA and its mutant variant, G4mut‐crRNA with four G mutations, across a PDS concentration of 0.5 µm and a KCl gradient from 0 to 100 mm. d) Molecular docking simulation results of LbCas12a paired with classical crRNA (left), G4‐crRNA (center), and G4mut‐crRNA (right). The red regions represent the RuvC domain of LbCas12a. The cyan regions denote the DR region in the crRNA sequence, while the dark blue regions correspond to the Spacer Region within the crRNA. The orange regions indicate the telomeric RG4 sequence present in G4‐crRNA, and the purple regions highlight the mutated telomeric RG4 sequence in G4mut‐crRNA. The results depict a shift in the interaction sites between the DR region and the protein in G4‐crRNA. The presence of the RG4 structure induces a significant conformational change in the protein. e) A bar graph illustrating the inhibition and subsequent recovery of G4‐crRNA *trans*‐cleavage activity in the presence of 0.5 µm PDS and 50 mm KCl, as the concentration of exogenous Telo G4 increases. Error bars are standard deviation (SD) (*n* = 3). f) The Michaelis–Menten kinetic curves illustrate the relationship between LbCas12a reaction velocity and substrate concentration under different conditions. g) Comparison of the Michaelis–Menten kinetic parameters of LbCas12a utilizing various crRNAs under different conditions.

To investigate our hypothesis, we designed a crRNA targeting a randomly synthesized DNA sequence (RS target) with a classical telomeric RG4 sequence (GGGUUA)_3_GGG at the 5 ′end (termed G4‐crRNA) and its canonical crRNA (RS crRNA, RS G4‐crRNA listed in Table S1, Supporting Information). We evaluated the *trans‐*cleavage activity of LbCas12a in the presence of varying concentrations of potassium chloride (KCl) and pyridostatin (PDS)^[^ ^51^ ^]^ by monitoring the increasing fluorescence signal. We input equal amounts of G4‐crRNA or classical crRNA, target DNA, LbCas12a, and F‐Q reporter (Table S1, Supporting Information, a single‐stranded DNA labeled with a fluorophore at one end and a quencher at the other which can be digested by activated LbCas12a to release fluorescence) along with different concentrations of potassium ions and PDS. PDS and potassium ions have been previously established as effective stabilizers of telomeric RG4, promoting the formation of stable RG4 structures.^[^ ^51^ ^]^ In the absence of PDS and potassium ions, the fluorescence emitted by the G4‐crRNA was slightly lower compared to the classical crRNA but was relatively similar (**Figure** **1**b; Figure S1, Supporting Information). This suggests that adding an RG4 at the 5′ end of crRNA does not significantly impact the LbCas12a activity. However, with increasing concentrations of PDS, a strong inhibition of LbCas12a activity was observed with G4‐crRNA. Classical crRNA fluorescence remained almost unaffected at PDS concentrations ranging from 0.25 to 0.5 µm, further indicating that stabilized RG4 at the 5′ end of crRNA can effectively repress LbCas12a activity (Figure 1b; Figure S1, Supporting Information). That is, we could suppress LbCas12a activity by designing a crRNA with an RG4 at the 5′ end and stabilizing the RG4 structure with RG4 stabilizers such as PDS.

To verify the ability of RG4 stabilizers to modulate the structure of G4‐crRNA, the impact of 0.5 µm PDS and a range of potassium ion concentrations (0–100 mm) on G4‐crRNA was systematically analyzed (Figure 1c). PAGE analysis confirmed that crRNA with telomeric RG4 formed a stable secondary structure under 0.5 µm PDS. The mutation of four Gs in the G4 sequence (G4mut‐crRNA), serving as a negative control, further validated the structural change of only RG4 under the influence of PDS, consistent with previous research.^[^ ^51^ ^]^ Furthermore, we employed circular dichroism (CD) spectroscopy to provide a more direct verification that G4‐crRNA can form a stable RG4 structure under PDS conditions, whereas G4mut‐crRNA does not exhibit this capability (Figure S2a–d, Supporting Information). To explore the potential molecular mechanisms underlying the inhibition of LbCas12a activity by the RG4 structure in the scaffold domain, we conducted molecular docking simulations. Using LbCas12a as the receptor and crRNA, G4‐crRNA, and G4mut‐crRNA as ligands, we utilized published PDB files^[^ ^55^ ^]^ or AlphaFold 3‐predicted PDB structures^[^ ^56^ ^]^ and performed calculations with the HDOCK^[^ ^57^ ^]^ and HNADOCK^[^ ^58^ ^]^ servers. The results showed that G4mut‐crRNA, which lacks the RG4 structure, binds to the LbCas12a RuvC domain similarly to classical crRNA, without significant structural changes, thus allowing normal function. In contrast, G4‐crRNA, which features the RG4 structure, fails to bind to the original protein binding site in the DR region. And the structure undergoes significant changes (Figure 1d). This suggests that the RG4 structure in the scaffold domain inhibits LbCas12a activity by disrupting its normal interaction with crRNA. Given the diversity and complexity of RG4 structures, we further investigated the generality of RG4‐mediated inhibition of LbCas12a activity by introducing several common RG4 structures—such as those from ‐KRAS, VEGF, and c‐kit^[^ ^23^ ^]^—at the 5′ end of crRNA. These structures consistently inhibited LbCas12a activity, indicating that the introduction of RG4 at the 5′ end of crRNA is a broadly applicable strategy for regulating LbCas12a activity (Figure S3, Supporting Information).

Subsequently, we hypothesized that adding exogenous telomeric DNA G‐quadruplex (GGGTTA)_3_GGG (Telo G4)^[^ ^1^, ^6^, ^10^ ^]^ could compete off PDS and potassium ions from G4‐crRNA,^[^ ^8^, ^10^ ^]^ thereby restoring LbCas12a activity (Figure 1a). The proposed mechanism is based on the fact that Telo G4 contains a similar G4 sequence as G4‐crRNA but is present in significantly higher concentrations. This allows Telo G4 to effectively chelate the free PDS and potassium ions in the solution, disrupting the stable RG4 structure^[^ ^59^ ^]^ initially formed by G4‐crRNA, which in turn reactivates the activity of the Cas protein. We tested this by measuring fluorescence recovery under the conditions of PDS concentration at 0.5 µm and KCl concentration ranging from 0 to 100 mm, with different concentrations of added Telo G4 (Figure 1e; Figures S3 and S4a–e, Supporting Information). Notably, our results showed that the fluorescence intensity gradually increased with the increase of Telo G4. Specifically, at 500 nm of Telo G4, the fluorescence intensity reached its peak values of 95.7% (PDS 0.5 µm, KCl 25 mm, Figure S4a, Supporting Information) and 94.7% (PDS 0.5 µm, KCl 50 mm, Figure 1e) fluorescence intensity recovery rates, respectively. However, further increases in Telo G4 led to a slight decrease in fluorescence, possibly due to an excessive amount of single‐stranded DNA (Telo G4) competing with F‐Q reporter thereby reducing the efficiency of *trans‐*cleavage activity of activated LbCas12a for F‐Q. At 100 mm KCl and 0.5 µm PDS, Telo G4 could only restore fluorescence to a maximum of 63.8%, this may indicate that high concentrations of potassium ions may hinder the competition of Telo G4 with the stabilizing G4‐crRNA (Figure S4a, Supporting Information).

To investigate the role of Telo G4 in inhibiting G4‐crRNA by RG4 stabilizers, we conducted control experiments using a single‐stranded Telo G4 sequence, a double‐stranded Telo G4 sequence, and a single‐stranded Telo G4 mutant sequence with four G mutations (Figure S4f, Supporting Information). The results indicate that only the addition of the single‐stranded Telo G4 sequence can restore the original function of G4‐crRNA inhibited by RG4 stabilizers; the double‐stranded structure or G4 variant of the non‐G4 structure cannot. Additionally, we utilized native PAGE to examine the gradual restoration of the stable G4‐crRNA secondary structure to the original non‐G4 structure with increasing single‐stranded Telo G4 sequence (Figure S2e, Supporting Information). This demonstrates that an excess of single‐stranded Telo G4 can counteract the RG4 stabilizers in RG4 and restore the original structure of G4‐crRNA.

### 2.2 G4‐crRNA‐Assisted CRISPR‐Cas12a System's *cis* and *trans*‐Cleavage Activity

First, we focused on the *cis‐*cleavage activity of the CRISPR‐Cas12a system assisted by G4‐crRNA in the presence of a FAM‐labeled Epstein‐Barr Virus (EBV) target (Table S1, Supporting Information). Specifically, we designed three different crRNA: EBV G4‐crRNA with an RG4 motif at the 5′ end, EBV G4mut‐crRNA featuring four mutations in the RG4 site acting as a control, and the standard EBV crRNA for comparison (Table S1, Supporting Information). Through the agarose gel analysis, we confirmed similar effective *cis‐*cleavage activity among EBV G4‐crRNA, EBV G4mut‐crRNA, and EBV crRNA (Figure S5a, lanes 2–4, Supporting Information). However, upon the introduction of 0.5 µm PDS and 50 mm KCl, the *cis‐*cleavage activity facilitated by EBV G4‐crRNA was nearly entirely inhibited (Figure S5a, lane 7, Supporting Information), while the controls, EBV crRNA (lane 5) and EBV G4mut‐crRNA (lane 6), maintained robust *cis‐*cleavage activity. This indicates that RG4 stabilizers effectively suppress CRISPR‐Cas12a's *cis‐*cleavage activity in crRNAs with RG4 structures without affecting traditional crRNA or G4 mutant sequences. Further investigation with the addition of 500 nm Telo G4 demonstrated a significant recovery of *cis‐*cleavage activity suppressed by the RG4 stabilizers, with a *cis*‐cleavage of target recovery rate peaking at 95.3% at 500 nm Telo G4 (Figure S5b,c, Supporting Information). This suggests that added Telo G4 can effectively counteract the G4 stabilizers' effects on G4‐crRNA‐assisted CRISPR‐Cas12a activity.

Then, to test the *trans‐*cleavage activity of the G4‐crRNA‐assisted CRISPR‐Cas12a system, we used a similar model with FAM‐free EBV target instead and additionally added F‐Q reporter as a reporter for LbCas12a's *trans‐*cleavage activity. We observed that modifications at the 5′ end of crRNA, whether it was EBV G4‐crRNA or EBV G4mut‐crRNA, did not diminish LbCas12a activity compared to the standard EBV crRNA (Figures S5d,e, Supporting Information). In the presence of RG4 stabilizers (0.5 µm PDS and 50 mm KCl), the fluorescence intensity generated by *trans‐*cleavage facilitated by EBV G4‐rRNA was reduced to 1.9% only, while crRNAs without the potential to form G4 structures retained more than 95% (Figure S5e, Supporting Information). This reduction might be due to the stabilizers promoting the formation of the RG4 structure, thereby impeding the interaction between LbCas12a and the crRNA sequence. Comparing fluorescence curves before and after the addition of G4 stabilizers, and with the subsequent introduction of 500 nm Telo G4, we concluded that Telo G4 can significantly restore the *trans‐*cleavage activity suppressed by the RG4 stabilizers, reaching a fluorescence intensity recovery rate of 90.2%. These findings elaborate on the nuanced interactions between the structural components of CRISPR‐Cas12a systems and their modulatory elements.

Furthermore, we studied the Michaelis–Menten kinetics of LbCas12a (Figure 1f,g; Figure S6, Supporting Information) and found that, when activated by target DNA, the catalytic efficiency (k_cat_/K_M_) of LbCas12a with regular crRNA was 5.36 × 10^5^ M^−1^ S^−1^. Adding 0.5 µm PDS and 50 mm KCl to the system reduced slightly the catalytic efficiency to 2.87 × 10^5^ M^−1^ S^−1^, while G4‐crRNA exhibited an efficiency of 3.59 × 10^5^ M^−1^ S^−1^, which both maintained effective *trans*‐cleavage activity. However, in the presence of 0.5 µm PDS and 50 mm KCl, G4‐crRNA's efficiency significantly dropped to 2.77 × 10^2^ M^−1^ S^−1^, demonstrating a marked reduction in *trans*‐cleavage activity. Upon adding 0.5 µm TeloG4, the catalytic efficiency was restored to 1.82 × 10^5^ M^−1^ S^−1^, reactivating the original effective *trans*‐cleavage activity.

**Figure 2:**
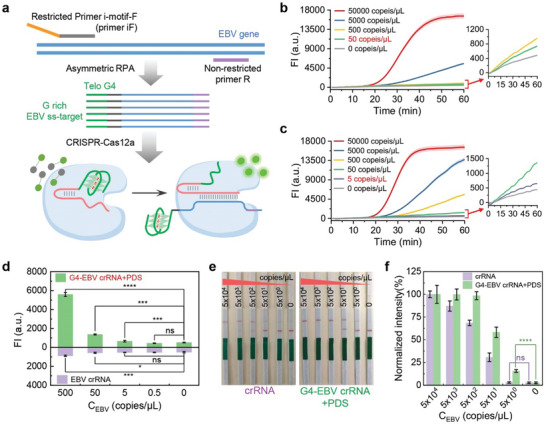
a) Schematic of nucleic acid detection using an asymmetric RPA‐G4‐crRNA assisted CRISPR‐Cas12a system. The restricted primer i‐motif‐F (primer iF) features a Telo G4 complementary sequence at its 5′ end. The non‐restricted primer, present at a concentration ten times that of primer iF, amplifies abundant target DNA containing single‐stranded Telo G4. This competes with G4 stabilizers to restore LbCas12a's detection activity, thus activating the CRISPR‐Cas12a system and releasing fluorescence. b) Real‐time fluorescence detection profiles of the CRISPR‐Cas12a system assisted by asymmetric RPA‐crRNA targeting EBV targets with different copy numbers, in the absence of PDS. Error bars are standard deviation (SD) (*n* = 3). c) Real‐time fluorescence detection profiles of the CRISPR‐Cas12a system assisted by asymmetric RPA‐G4‐crRNA targeting the EBV target with different copy numbers, in the presence of PDS. Error bars are standard deviation (SD) (*n* = 3). d) Comparison of fluorescence intensity bar graphs for EBV target copy number detection, assisted by asymmetric RPA‐G4‐crRNA (PDS+) and asymmetric RPA‐crRNA (PDS‐), respectively. Notes: ns, *p* > .05, *, *p* ≤ 0.05, **, *p* ≤ 0.01, ***, *p* ≤ 0.001, ****, *p* ≤ 0.0001. Error bars are S.D. (*n* = 3). e) Strip test results demonstrating the detection of different concentrations of EBV using asymmetric RPA‐crRNA (PDS‐) (left) and asymmetric RPA‐G4‐crRNA (PDS+) (right) assisted CRISPR‐Cas12a nucleic acid detection system. The images were captured using a smartphone. f) Quantitative analysis of the results shown in Figure 2e, performed using ImageJ. Notes: ns, *p* > 0.05, ****, *p* ≤ 0.0001. Error bars are S.D. (*n* = 3). Data were analyzed by two‐tailed *t‐test*.

Next, we developed an approach that combines asymmetric Recombinase polymerase amplification (RPA)^[^ ^60^ ^]^ with RG4‐assisted CRISPR‐Cas12a in a one‐pot assay (**Figure** **2**a). We compared the performance of traditional crRNA and G4‐crRNA in this assay, with and without PDS. We found that for detecting EBV, conventional crRNA (PDS‐), G4‐crRNA (PDS‐), and crRNA (PDS+) behaved similarly, all struggling to detect samples below 50 copies µL^−1^ due to template cleavage by CRISPR reaction during isothermal amplification. However, G4‐crRNA with PDS inhibited CRISPR activity during amplification, while asymmetric RPA generated numerous target sequences with single‐stranded G4 sequences (Telo G4), enabling reactivation of LbCas12a and detection of as low as 5 copies µL^−1^ (Figure 2b–d; Figure S7, Supporting Information).

In this approach, at the beginning of the asymmetric RPA, the function of CRISPR‐Cas12a assisted by G4‐crRNA was temporarily hindered by the presence of a stable RG4 structure at the 5′ end of crRNA induced by RG4 stabilizers. This is the reason why the CRISPR reaction did not disturb the template during the isothermal amplification. In addition, the newly introduced primer, Restricted Primer i‐motif‐F (primer iF), contained an i‐motif^[^ ^61^ ^]^ (complementary sequence to Telo G4), which could generate a substantial quantity of target DNA with single‐stranded Telo G4 during amplification. This competed with G4‐crRNA for RG4 stabilizer, thus restoring the original structure of G4‐crRNA. Moreover, the amplified target DNA could activate *trans‐*cleavage activity of LbCas12a, resulting in the breakdown of F‐Q reporter and the subsequent release of fluorescence (Figure 2a).

To verify that our designed asymmetric RPA effectively amplifies the template to achieve high concentrations of single‐stranded amplified target sequences, we analyzed the amplified products at various time points (0–60 min) using 3% agarose gel electrophoresis. This analysis confirmed that the asymmetric RPA successfully generates a sufficient quantity of single‐stranded target sequences (Figure S8, Supporting Information). Considering that double‐stranded structures can be unwound by associated proteins within the RPA buffer, it was crucial to assess whether the PDS‐stabilized RG4 structure at the 5′ end of G4‐crRNA remains relatively stable in the RPA mixture. We employed gel imaging to evaluate the structures of G4mut‐crRNA and G4‐crRNA formed under both the presence and absence of PDS and potassium ions in the RPA mixture. Our experiments demonstrated that, even under the conditions of the RPA buffer, PDS can effectively stabilize the formation of a relatively stable RG4 structure at the 5′ end of G4‐crRNA, thereby significantly suppressing the activity of LbCas12a (Figure S9, Supporting Information). Subsequently, we evaluated the performance of our newly developed asymmetric RPA‐Cas12a system with G4‐crRNA (PDS+) against the classical RPA‐Cas12a system with crRNA using clinical samples. We collected plasma samples from seven patients, four of whom were clinically diagnosed with positive EBV infection through qPCR, exhibiting high EBV viral loads, while the remaining three were diagnosed as negative, with low EBV copy numbers. We extracted DNA from these plasma samples and conducted tests using both the asymmetric RPA‐Cas12a system with G4‐crRNA (PDS+) and the classical RPA‐Cas12a system with crRNA. The experimental results showed that both our developed method and the classical method were consistent with clinical diagnosis (Figure S10, Supporting Information). However, it is important to note that the asymmetric RPA has inherent limitations, including lower amplification efficiency compared to the classical RPA. Consequently, while our asymmetric RPA‐Cas12a system with G4‐crRNA (PDS+) yielded comparable results to the classical RPA‐Cas12a system with crRNA in the detection of specific clinical samples, it did not demonstrate a significant advantage in this context.

To develop a rapid detection strategy, we combined the asymmetric RPA RG4‐assisted CRISPR‐Cas12a one‐pot method with lateral flow strip technology.^[^ ^28^, ^62^ ^]^ We replaced the F‐Q reporter DNA reporter in our initial experiments with a FAM‐biotin DNA reporter, which is labeled with biotin on one end and FAM on the other. Using commercially available lateral flow strips based on an immune‐chromatographic analysis system, we inserted the strips into the solution after the CRISPR isothermal reaction. Colloidal gold‐conjugated anti‐FAM antibodies bind to either intact or cleaved FAM‐biotin reporters. This complex then interacts with solid phases containing streptavidin (control line, C) and anti‐anti‐FAM (test line, T). We captured images using a smartphone for qualitative and quantitative analysis. Our method demonstrated superior detection limits compared to traditional approaches, with a sensitivity down to 5 copies µL^−1^, whereas classical methods only detected down to 50 copies µL^−1^ (Figure 2e,f).

This design is free of chemical modifications, eliminating the need to modify the crRNA sequence in advance or carry out additional steps to reactivate the Cas12a enzyme. However, it is worth noting that this design relies on asymmetric amplification, which comes with inherent advantages and limitations found in all asymmetric amplification methods, such as completely free from PAM limitations^[^ ^63^ ^]^ and lower amplification efficiency compared to standard isothermal amplification.^[^ ^64^ ^]^

### 2.3 Universal G4‐DR Design for Detection of Various Nucleic Acid Targets

**Figure 3:**
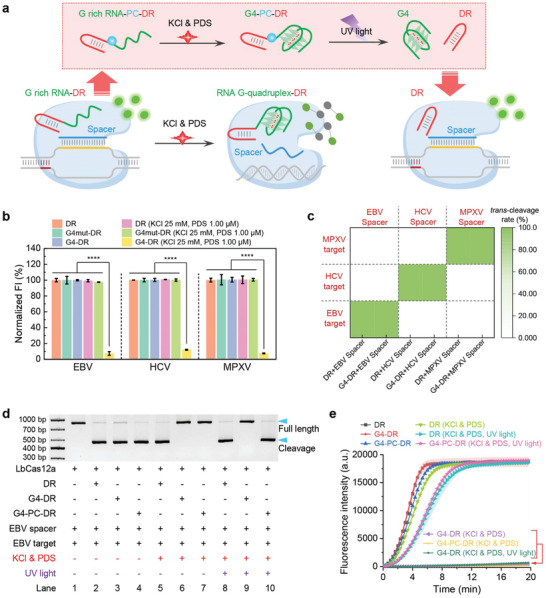
a) The schematic diagram illustrates the inhibition of Cas12a activity by the RG4 sequence present at 5′ end of DR region along with RG4 stabilizer. The versatility of this inhibition method is highlighted by its applicability to various target sequences by simply changing spacer regions. The presence of a PC linker between the RG4 sequence and the DR region does not impede the inhibition of Cas12a activity as the stable RG4 at the 5′ end of the DR region remains intact. Exposure to UV light triggers the cleavage of the PC linker, leading to the separation of RG4 from the DR region, consequently restoring the activity of Cas12a. b) Comparison of fluorescence bar graphs demonstrate the response of the RG4 stabilizer in classic DR and RG4 sequences containing DR (G4‐DR), as well as its mutant G4mut‐DR (where four guanines are mutated, preventing RG4 structure formation) across different spacer regions targeting EBV, HCV, and MPXV. Error bars are standard deviation (SD) (*n* = 3). c) Heat map analysis demonstrating target specificity. The heat map illustrates the binding specificity of G4‐DR and classical DR when matched with EBV spacer for the detection of EBV target, HCV spacer for the detection of HCV target, and MPXV spacer for the detection of MPXV target. The color gradient represents the relative *trans‐*cleavage rate levels, calculated from original fluorescence data. d) The agarose gel image displays the *cis‐*cleavage activity toward targeted nucleic acids for classic DR, G4‐DR, and G4‐PC‐DR in the presence and absence of the RG4 stabilizer, with or without UV light exposure. e) Real‐time fluorescence graphs compare the *trans‐*cleavage activity toward targeted nucleic acids of classic DR, G4‐DR, and G4‐PC‐DR, with and without the RG4 stabilizer, under conditions of UV illumination or no illumination. Error bars are standard deviation (SD) (*n* = 3).

Based on the findings of Shebanova et al.,^[^ ^65^ ^]^ J. Moon et al.,^[^ ^40^ ^]^ and Y. Chen et al,^[^ ^66^ ^]^ have shown that splitting the crRNA of Cas12a into a universal DR region and replaceable Spacer region enables detection of different nucleic acid targets with adjustments to the Spacer region only. Expanding upon this, we hypothesized that incorporating the RG4 sequence at the 5′ end of the DR region, we anticipate improved control and specificity in targeting different nucleic acid sequences. This proposed modification has the potential to streamline the process of designing Cas12a‐based assays for a wide range of applications in molecular biology and diagnostics (**Figure** **3**a).

To test our hypothesis, we designed a DR with a classic telomeric RG4 sequence at the 5′ end (G4‐DR), as well as a G4mut‐DR with four G mutations in the RG4 sequence, and a classic DR. We then evaluated their *trans‐*cleavage activity of LbCas12a by measuring the released fluorescence under varying concentrations of KCl and PDS. Under conditions without PDS and potassium ions, the G4‐DR displayed fluorescence values similar to the classic DR, indicating that the addition of an RG4 at the 5′ end of the DR did not significantly affect the LbCas12a activity. However, as the concentrations of PDS and potassium ions increased, the G4‐DR showed strong inhibition of LbCas12a activity, with inhibition rates often exceeding 90%. Notably, at PDS 0.5 µm, KCl 100 mm, and PDS 1 µm, KCl 50 or 100 mm, the classic DR exhibited minimal inhibition of fluorescence values, while the G4‐DR demonstrated significant inhibition, further confirming that the introduction of an RG4 at the 5′ end of the DR can effectively modulate LbCas12a activity (Figure S11a,b, Supporting Information). Further experiments using native PAGE confirmed that the DR sequence, which includes the telomeric RG4 motif, could establish a stable secondary structure in the presence of 0.5 µm PDS and 100 mm KCl, or 1.0 µm PDS and 25–50 mm KCl (Figure S11c, Supporting Information). These results provide additional evidence supporting the critical role of the telomeric RG4 motif at the 5′ end of the DR in influencing LbCas12a activity. In contrast, the G4mut‐DR, a similar control that is unable to form the RG4 structure, maintained its original structure, highlighting the secondary structure alterations induced by the telomeric RG4 in the presence of PDS and potassium ions.

Subsequent experiments involved substituting different Spacer regions to confirm the adaptability of G4‐DR to various nucleic acid targets. By pairing G4‐DR, G4mut‐DR, and classic DR with EBV, Hepatitis C virus (HCV), or Monkeypox virus (MPXV) Spacer regions, we demonstrated that all three targets could be effectively detected with comparable fluorescence intensity and *trans‐*cleavage rate. Under conditions of PDS 1 µm and KCl 25 mm, the G4‐DR assisted CRISPR‐Cas12a exhibited strong inhibition of *trans‐*cleavage activity toward EBV, HCV, and MPXV targets. Conversely, G4mut‐DR and classic DR were unaffected by the RG4 stabilizer (Figure 3b,c; Figures S12 and S13, Supporting Information). These findings underscore the potential of utilizing RG4 stabilizers to modulate the universal G4‐DR assisted LbCas12a activity, allowing for applications to different nucleic acid targets through matching with specific Spacer regions (Figure 3c; Figure S12 and S13, Supporting Information).

### 2.4 Photo‐Regulation of Cas12a Function with G4‐PC‐DR

To achieve precise photo‐regulation of Cas12a function and address the inherent incompatibility between isothermal amplification and CRISPR reaction in the isothermal amplification‐CRISPR‐Cas12a nucleic acid detection system, we hypothesized that the incorporation of a commercially available PC linker between RG4 and DR (G4‐PC‐DR) could still inhibit LbCas12a's cleavage activity with the aid of a G4 stabilizer. Upon complete isothermal amplification in a single tube, simple UV light exposure would lead to cleavage of the PC linker, detaching the RG4 structure from the 5′ end of the DR region and thereby restoring LbCas12a's functionality.

To test our hypothesis, we compared the *trans* and *cis‐*cleavage activities of CRISPR‐Cas12a assisted by G4‐PC‐DR, G4‐DR, and the classic DR. We first conducted UV exposure experiments and confirmed that the PC linker was completely cleaved under UV light (365 nm, 35 W) within 30 s, disconnecting the RG4 and reverting G4‐PC‐DR to the typical DR. (Figure S14, Supporting Information).

Subsequently, we demonstrated that G4‐PC‐DR, like G4‐DR and classic DR, exhibited effective *trans* and *cis‐*cleavage activities. Under conditions of 1 µm PDS and 25 mm KCl, both G4‐PC‐DR and G4‐DR assisted LbCas12a showed strong inhibition of their *trans* and *cis‐*cleavage activities, while the classic DR was unaffected. Finally, we successfully restored LbCas12a activity by exposing G4‐PC‐DR to UV light (365 nm, 35 W) for 30 s, effectively eliminating the inhibition (Figure 3d,e).

### 2.5 G4‐PC‐DR Assisted CRISPR‐Cas12a System for Enhanced Sensitivity in Clinical EBV Infection Detection

**Figure 4:**
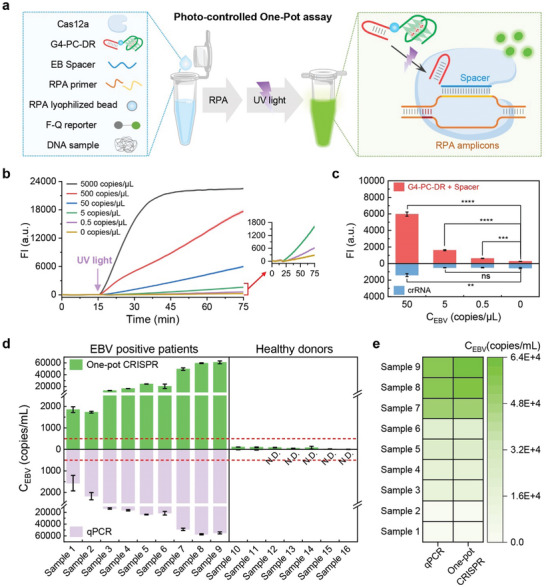
a) The schematic illustrates the G4‐PC‐DR Assisted CRISPR‐Cas12a System, designed for enhanced sensitivity in clinical sample detection. b) Real‐time fluorescence graphs depict the detection strategy for different concentrations of EB target. The presence of an RG4 stabilizer causes the G4‐PC‐DR configuration to inhibit LbCas12a activity. Following 15 min of RPA and 30 s of UV light exposure, the RG4 dissociates from the DR region, restoring LbCas12a activity. This innovation resolves the compatibility issue between isothermal amplification and CRISPR activity, enabling the detection of a single copy of EBV. Error bars are standard deviation (SD) (*n* = 3). c) A comparison between the G4‐PC‐DR assisted CRISPR‐Cas12a System and the traditional crRNA‐assisted CRISPR‐Cas12a System reveals that the former can detect EBV as low as 0.5 copies µL^−1^, while the latter struggles with 5 copies µL^−1^. Notes: ns, *p* > 0.05, *, *p* ≤ 0.05, **, *p* ≤ 0.01, ***, *p* ≤ 0.001, ****, *p* ≤ 0.0001. Error bars are S.D. (*n* = 3). Data were analyzed by two‐tailed *t‐test*. d) Quantitative analysis of EBV in blood samples from 9 EBV‐infected patients and 7 healthy individuals is conducted using our G4‐PC‐DR assisted CRISPR‐Cas12a System and the classical qPCR method. Notes: N.D., Not detected, Error bars are standard deviation (SD) (*n* = 3). e) A heatmap representation showcases the EBV load in blood samples from 9 EBV‐infected patients, comparing our one‐tube method with the classical qPCR approach.

Building upon the advancements previously mentioned, we have engineered a novel CRISPR‐Cas12a nucleic acid detection system. This innovative approach incorporates G4‐PC‐DR, designed to match alongside RPA technology, enabling the detection of various nucleic acid targets via adding different Spacer region. In the presence of G4 stabilizers, G4‐PC‐DR successfully inhibits LbCas12a activity. Following a 15‐min isothermal amplification at 37 °C, a brief 30‐s ultraviolet light exposure induces a separation between DR and RG4, initiating the CRISPR‐Cas12a reaction. This reaction digests the F‐Q reporter, emitting a fluorescent signal. The concentration of nucleic acid targets can be effectively monitored in real‐time through this emitted fluorescence (**Figure** **4**a).

For illustration, our system's proficiency in detecting lower concentrations of DNA targets, as exemplified with EBV detection, surpasses that of the classical EBV crRNA. Before amplification, LbCas12a's *cis‐*cleavage activity degrades the EBV target, eluding detection under 50 copies µL^−1^ by the classical EBV crRNA method (Figure 4c; Figure S15). Our system, conversely, is capable of identifying 0.5 copies µL^−1^, underscoring its significantly enhanced sensitivity over traditional crRNA methodologies (Figure 4b,c; Figure S15, Supporting Information).

To evaluate the applicability of our newly developed G4‐PC‐DR assisted PRA‐CRISPR One‐pot nucleic acid detection system, initially designed for LbCas12a, we investigated its effectiveness with other Cas12a variants, including ArCas12a, AsCas12a, ErCas12a, and FnCas12a. We first assessed the inhibitory effects of G4‐PC‐DR on these variants under conditions involving PDS and potassium ions. Our results showed that PDS‐stabilized G4‐PC‐DR effectively inhibited the activity of these variants, similarly to LbCas12a, and that their activity was restored upon exposure to ultraviolet light (Figure 3e; Figure S16, Supporting Information).

Next, we applied the same conditions originally established for LbCas12a, substituting it with other Cas12a variants within our photo‐controlled G4‐PC‐DR assisted PRA‐CRISPR One‐pot nucleic acid detection system. It is noteworthy that all the variants demonstrated the capacity to detect EBV concentrations as low as 0.5 copies µL^−1^, a level comparable to that observed with LbCas12a despite slight fluctuations in fluorescence intensity were observed across the different variants (Figure 4b; Figure S17, Supporting Information). These findings highlight the universality and robustness of our detection system in accommodating multiple Cas12a variants.

Furthering our investigation, we applied our method to detect clinical EBV infections in blood samples. Given EBV's association with numerous human cancers, such as nasopharyngeal carcinoma, epithelial carcinoma, and lymphoid diseases, its detection holds substantial importance in medical diagnostics. We obtained nine blood samples from patients afflicted with EBV and seven samples from healthy donors for further validation. DNA extracted from these samples was analyzed using both the conventional method and our newly developed approach. Employing a concentration standard curve established with EBV nucleic acid target standards, we calculated the copy numbers of the EBV in clinical samples using our light‐activated one‐pot CRISPR‐Cas12a detection method and compared these results to those obtained via classical qPCR (Figure 4d,e; Figures S18 and S19, Supporting Information). Our findings reveal that our method not only possesses a lower detection limit but also exhibits sensitivity comparable to that of traditional qPCR. Requiring only isothermal detection and a markedly shorter assay time (≈30–40 min), our approach provides significant detection advantages, especially in scenarios of low sample volume and limited equipment availability.

## 3 Conclusion

In conclusion, our study introduces a unique secondary structure of RG4 at the 5′ end of crRNA that addresses the significant challenge of precisely controlling Cas12a activity without the need for chemical modification to enhance nucleic acid detection. Other secondary structures, such as theophylline RNA aptamer at the 5′ end of crRNA, do not possess the same capabilities (Figure S20, Supporting Information).

First, our results show that the addition of RG4 to the 5′ end of crRNA or the DR region does not significantly affect the cleavage activity of Cas12a, while the presence of G4 stabilizer (potassium ions and PDS) promotes the formation of a stable RG4 structure, effectively inhibiting Cas12a activity. This inhibition can be reversed by the introduction of ss DNA G4 sequence, thereby restoring the activity of Cas12a.

Subsequently, we have developed a novel method for nucleic acid detection that combines asymmetric RPA with RG4‐assisted CRISPR‐Cas12a in a single reaction. Comparative analysis of traditional crRNA and G4‐crRNA demonstrates that G4‐crRNA with PDS can suppress CRISPR activity, while the ss G4 sequence generated by asymmetric RPA can reactivate Cas12a, enabling detection of samples with as few as 5 copies µL^−1^. Importantly, this design does not require chemical modifications or additional steps to reactivate Cas12a enzyme.

Furthermore, by incorporating a PC linker between RG4 and the DR region (G4‐PC‐DR), we have established a universal light‐controlled system that allows controlled activation of Cas12a for any target by simply changing the replaceable Spacer region. This approach not only circumvents the inherent incompatibility between isothermal amplification systems and CRISPR reactions but also offers a cost‐effective and less complex solution for nucleic acid detection. Our approach benefits from previously developed UV‐controlled isothermal amplification‐CRISPR‐Cas12a detection methods^[^ ^38^, ^39^, ^46^, ^67^, ^68^ ^]^ and split crRNA strategy,^[^ ^40^, ^65^, ^66^ ^]^ while offering enhanced universality, ease of use, and practical scalability compared to earlier UV‐controlled techniques. By preparing only the G4‐PC‐DR, our system can be customized to match any Spacer for the detection of various targets, thereby streamlining the process and reducing detection costs (Figure S21, Supporting Information).

The development of the G4‐PC‐DR assisted CRISPR‐Cas12a system not only simplifies the process of nucleic acid detection by eliminating the need for separate tubes and reducing the risk of contamination but also enhances the sensitivity of clinical EBV detection. This breakthrough has far‐reaching implications, opening new avenues for the application of CRISPR‐based technologies in clinical diagnostics, therapeutic interventions, and beyond.

In conclusion, our study demonstrates the potential of utilizing RG4 structural elements to regulate Cas12a enzyme activity with precision, thereby paving the way for the development of more efficient, accurate diagnostic tools. This represents a significant finding, with the potential to enhance our ability to diagnose diseases with precision and efficacy.

## 4 Experimental Section

### Chemicals and Materials

All chemicals were purchased from Macklin (Shanghai, China), except where otherwise specified. LbCas12a protein (Cat No. CAS12‐010B) was expressed and purified from *E. coli* by EZassay Biotech (Shenzhen, China). RNA and DNA were synthesized and purified by Shanghai Generay Biotech Co., Ltd (Shanghai, China) or Guangzhou IGE Biotechnology Ltd (Guangzhou, China). All experimental consumables, including PCR tubes/plates, cell culture dishes/plates, and filter universal pipette tips, were procured from NEST Biotechnology Co., Ltd. (Wuxi, China). RT‐RPA 2.0 (Cat No. 901221021) and lateral flow detection paper trips (Cat No. CAS‐cmCSA01) were supplied from Keer Life (Suzhou, china). T7 RNA polymerase (Cat No. DD4101‐01), Murine RNase inhibitor (Cat No. R301), Pyrophosphatase Inorganic (Cat No. DD4103‐0 PC), VAHTS RNA Clean Beads (Cat No. N412‐02) were obtained from Vazyme Biotech Co., Ltd (Nanjing, China). GelstainRed Nucleic Acid Dye (Cat No. S2009L) was purchased from US EVERBRIGHT (Suzhou, China). Hieff Canace Plus High‐Fidelity DNA Polymerase (Cat No. 10153ES76), MolPure Cell/Tissue DNA Kit (Cat No. 18700ES70), Proteinase K solution (Cat No. 10412ES76) and MolPure PCR Purification Kit (Cat No. 19106ES70) were bought from Yeasen Biotechnology Co., Ltd. (Shanghai, China). QIAamp DNA Blood Midi Kit (Cat No. 51183) were bought from Qiagen (USA). Ready to use 2xTaq Master Mix (Cat No. GK8005) was provided from Shanghai Generay Biotech Co., Ltd. Ribonuclease Inhibitor (AI101) was bought from TransGen Biotech (Beijing China). ApexHF HS DNA Polymerase FS (Cat No. AG12201) was purchased from Accurate Biotechnology (Hunan) Co., Ltd. (Changsha, China). AsCas12a (Cat No. CS12AC050) was purchased from Synbio Technologies (Suzhou, China). ErCas12a (Cat No. Z03762‐100) was bought from GenScript Biotech Corporation (Nanjing, China). FnCas12a (Cat No. D0510S) was obtained from Beyotime (Shanghai, China). ArCas12a (Cat No. 14702ES65) was provided by Yeasen Biotechnology Co., Ltd. (Shanghai, China).

### Inhibition and Recovery Assay for CRISPR‐Cas12a's *trans‐*Cleavage Activity Using G4‐crRNA

First, the 10× LbCas12a Reaction Buffer was prepared, which consisted of 100 mm Tris‐HCl (pH 8.5), 100 mm MgCl_2_, 10 mm DTT, 400 mm Glycine, and 0.1% (v/v) Tween 20. This buffer was stored at −20 °C for future use. For the inhibition assay, 2 µL of crRNA or G4‐crRNA (including telo‐crRNA, KRAS‐crRNA, VEGF‐crRNA, ckit‐crRNA) or G4mut‐crRNA (50 nm, Table S1, Supporting Information) was pre‐incubated at room temperature with a combination of concentration‐gradient PDS and KCl, adding Milli‐Q water to achieve a final volume of 10 µL in a 0.2 mL microcentrifuge tube for 10 min. Subsequently, 1 µL of the ds target (20 nm), 1 µL of the F‐Q reporter (5 µm), 1 µL of LbCas12a (2 µm), and 2 µL of the 10× LbCas12a Reaction Buffer were added. The volume was further adjusted with Milli‐Q water to attain a final volume of 20 µL. The resulting mixture was incubated at 37 °C in a YEASEN Celemetor Real‐Time Fluorescent Quantitative PCR Analysis System (Celemetor‐96, Yeasen Biotechnology Co., Ltd., Shanghai, China) for 20 min, during which fluorescence readings were recorded every 20 s.

For the recovery assay, 2 µL of the aforementioned RNA was pre‐incubated under the same conditions. Afterward, concentration‐gradient ss or ds Telo G4 (0, 15.6, 31.2, 62.5, 125, 250, 500, 1000 nm, Table S1, Supporting Information) was added, and incubation continued for an additional 10 min. Finally, the LbCas12a reaction was conducted, and fluorescence data were recorded in the same manner as described above.

### Non‐Denaturing PAGE of the Formation of G‐Quadruplex (G4) by G4‐crRNA and G4‐DR

FAM‐labeled RNAs (G4‐crRNA‐FAM, G4mut‐crRNA‐FAM, G4‐DR‐FAM, G4mut‐DR‐FAM, 80 nm, Table S1, Supporting Information) were annealed in a buffer (10 mm Tris‐HCl, pH 7.4) containing various concentrations of KCl and PDS. After incubation, the samples were analyzed using non‐denaturing 12% PAGE. RNA oligomers were visualized through scanning with Bio‐Rad ChemiDoc Imagers after electrophoresis.

### Non‐Denaturing PAGE of Competitive Reaction Experiments for G4‐crRNA and Telo G4

FAM‐labeled RNAs (G4‐crRNA‐FAM, G4mut‐crRNA‐FAM, 80 nm, Table S1, Supporting Information) were annealed in a buffer (10 mm Tris‐HCl, pH 7.4) containing KCl (50 mm) and PDS (0.5 µm), and various concentrations of Telo G4 (0, 125, 250, 500, 1000, 2000, 4000 nm, Table S1, Supporting Information). After incubation, the samples were analyzed using non‐denaturing 12% PAGE. The gel was visualized through scanning with Bio‐Rad ChemiDoc Imagers after electrophoresis.

### Inhibition and Recovery Assay for CRISPR‐Cas12a's *cis‐*Cleavage Activity Using G4‐crRNA

For the inhibition assay, pre‐incubated 2 µL of EBV crRNA, EBV G4‐crRNA, or EBV G4mut‐crRNA (100 nm, Table S1, Supporting Information) at room temperature with 1 µL of PDS (5 µm) and 2 µL of KCl (250 mm) in a 0.2 mL microcentrifuge tube for 10 min. Following this, added 1 µL of FAM‐labeled ds EBV target (200 nm), 1 µL of LbCas12a (1 µm), and 1 µL of the 10× LbCas12a Reaction Buffer. Adjusted the final volume to 10 µL with Milli‐Q water. The resulting mixture was incubated at 37 °C for 30 min. After this incubation, added 40 µg of Proteinase K and continued incubation at 37 °C for an additional 15 min to digest protein for further gel electrophoresis.

For the recovery assay, 2 µL of the above RNA was pre‐incubated under the same conditions. A concentration gradient of ss Telo G4 (0, 15.6, 31.2, 62.5, 125, 250, 500, 1000 nm, Table S1, Supporting Information) was then added and incubated for another 10 min. The subsequent LbCas12a reaction should be performed as described above. Finally, LbCas12a *cis*‐cleavage activity was analyzed by 2% agarose gel electrophoresis at 120 V for ≈30 min.

### Molecular Docking Simulations

To conduct the molecular docking simulations, three sets of PDB files were prepared. Initially, the LbCas12a structure (PDB code: 6NME)^[^ ^55^ ^]^ was downloaded from the RCSB PDB database. Using PyMOL, 6NME structure was processed to obtain separate PDB files for the LbCas12a protein and the crRNA. For the RG4 structure, AlphaFold3 was employed by inputting the RG4 sequence.^[^ ^56^ ^]^ The predicted RG4 structure obtained from AlphaFold3 was then processed in PyMOL^[^ ^69^ ^]^ to generate its respective PDB file.^[^ ^56^ ^]^ Notably, the structure predicted by AlphaFold3 closely resembled the one designated with PDB code: 143D.^[^ ^70^ ^]^ To assemble the G4‐crRNA complex, the PDB files of crRNA and the predicted RG4 structure were imported into the HNADOCK server (http://huanglab.phys.hust.edu.cn/hnadock/), which performed the targeted docking and yielded the G4‐crRNA PDB file.^[^ ^58^ ^]^

Subsequently, docking experiments were set up where LbCas12a served as the receptor and crRNA, G4‐crRNA, and G4mut‐crRNA were each used as ligands. The respective PDB files were uploaded into the HDOCK server (http://hdock.phys.hust.edu.cn/)^[^ ^57^ ^]^ or alternatively used the AlphaFold3 server (https://alphafoldserver.com/) for the docking simulations.^[^ ^56^ ^]^ The resulting complexes were visualized and analyzed using PyMOL to assess the interactions and structural conformations.^[^ ^69^ ^]^

### Michaelis–Menten Kinetics Calculations

The Michaelis–Menten^[^ ^34^ ^]^ kinetics was measured following the protocol given by Ramachandran et al.^[^ ^71^ ^]^ The experimental procedures were as follows: 1) 5 nm of crRNA or G4‐crRNA was incubated with different concentrations of KCl and PDS at room temperature for 10 min. 2) The 50 nm or 1 µm of LbCas12a was mixed with the mixtures in Step 1 and then incubated with 1 nm of dsDNA activator in 1× LbCas12a reaction buffer at 37 °C for 20 min. Specifically, 1 µm of LbCas12a was incubated with G4‐crRNA, KCl, and PDS mixtures, 25 nm of LbCas12a was mixed with the other mixtures. 3) The *trans*‐cleavage assay was initiated by incubating the mixture from Step 2 with 31.25 nm, 62.5 nm, 125 nm, 250 nm, 500 nm, and 1 µm of the ssDNA reporters in 1× LbCas12a reaction buffer at 37 °C. The fluorescence readouts were obtained every 10 s and the first 600 s of the fluorescence data were used to calculate the initial reaction velocities. The fluorescence data used in the calculation was a result of the raw fluorescence data background‐subtracted by the average fluorescence intensities from four buffer‐only samples.

The calibration of fluorescence versus substrate concentration was carried out with concentrations of reporters varying from 31.25 nm to 1 µm, pre‐cleaved in a reaction containing 1×LbCas12a reaction buffer, 50 nm of LbCas12a, 25 nm of crRNA, and 10 nm of dsRNA activator. The reaction lasted 30 min at 37 °C, and at the end of the reaction the fluorescence values for all reporter concentrations were constant. A linear fitting between background‐subtracted fluorescence 𝐹_𝑐𝑙_ and cleaved reporter concentration 𝑐_𝑐𝑙_ was performed (Figure S1a, Supporting Information), given by 𝐹_𝑐𝑙_ = 63.77709 𝑐_𝑐𝑙_, where 𝑐_𝑐𝑙_ is in a unit of nm and 𝐹_𝑐𝑙_ is in the arbitrary fluorescence units (a.u.) of our thermal cycler. Similarly, the calibration of fluorescence versus uncleaved reporter concentration was performed (Figure S1b, Supporting Information). A linear fit was obtained between background‐subtracted fluorescence 𝐹_𝑢𝑐𝑙_ and uncleaved reporter concentration 𝑐_𝑢𝑐𝑙_, given by 𝐹_𝑢𝑐𝑙_ = 1.82727 𝑐_𝑢𝑐𝑙_, where 𝑐_𝑢𝑐𝑙_ is in a unit of nm and 𝐹_𝑢𝑐𝑙_ is in the fluorescence units (a.u.) of our thermal cycler.

Next, a mathematical model was established to estimate the reaction velocity based on the calibration curves. Theoretically, the background‐subtracted fluorescence 𝐹(𝑡) as a function of time should be contributed by fluorescence from both the cleaved reporters 𝐹_𝑐𝑙_(𝑡) and uncleaved reporters 𝐹_𝑢𝑐𝑙_(𝑡):

(1) $$F\left(t\right)=F_{cl}\left(t\right)+F_{ucl}\left(t\right)$$

The linear fitting results are as follows:

(2) $$F\left(t\right)=63.77709C_{cl}\left(t\right)+1.82727C_{ucl}\left(t\right)$$

With 𝑐_𝑐𝑙_(𝑡) + 𝑐_𝑢𝑐𝑙_(𝑡) = 𝑐_0_, where 𝑐_0_ is the initial concentration of uncleaved reporters, Equation (2) can be transferred to:

(3) $$\begin{array}{ccc} F(t) & = & 63.77709C_{cl}\left(t\right)+1.82727\left(C_{0}-C_{cl}\left(t\right)\right)\\ & & =61.94982C_{cl}\left(t\right)+1.82727C_{0} \end{array}$$

The reaction velocity 𝑑𝑐_𝑐𝑙_/𝑑𝑡 in nm s^−1^ is obtained by differentiating Equation (3) with respect to time as:

(4) $$\frac{dc_{cl}}{dt}=\frac{1}{61.94982}\;\times \frac{dF}{dt}$$

In this way, the reaction velocity in nm s^−1^ can be calculated by measuring the fluorescence enhancement rate in a.u./s.

The reaction velocity data for each substrate concentration were obtained and performed the Michaelis–Menten kinetics fitting using the following equation:^[^ ^71^ ^]^

(5) $$v=k_{cat}\;E_{0}\frac{\left[S\right]}{K_{M}+\left[S\right]}$$

where 𝑘_𝑐𝑎𝑡_ is the catalytic turnover rate of the enzyme, 𝐸_0_ is the activated enzyme concentration (25 and 500 nm in this experiment), [𝑆] is the substrate concentration, and 𝐾_𝑀_ is the Michaelis–Menten constant.

### One‐Pot Assay of Asymmetric RPA and CRISPR‐Cas12a Using EBV crRNA and G4‐EBV crRNA

The 10× RPA‐Cas12a buffer was prepared containing 0.1% Tween 20, 400 mm Glycine, 100 mm Tris‐HCl, 10 mm DTT, 400 mm KCl, and 324 mm MgCl_2_, adjusted to pH 8.5. This buffer was aliquoted and stored at −20 °C for single‐use to prevent any potential contamination. Each lyophilized RPA pellet (RT‐RPA 2.0, Cat No. 901221021, Keer Life, Suzhou, China) was reconstituted in 25 µL of Milli‐Q water while kept on ice. For each reaction, 10 µL of the RPA mixture was transferred to a pre‐cooled 0.6 mL microcentrifuge tube, along with the following components: 1 µL of EBV RPA‐imotif‐F (4 µm, Table S1, Supporting Information), 1 µL of EBV RPA‐R (40 µm, Table S1, Supporting Information), 1 µL of EBV crRNA or G4‐EBV crRNA (250 nm), 1 µL of the F‐Q reporter (5 µm), 1 µL of LbCas12a (2 µm), and 1 µL of the 10× RPA‐Cas12a buffer. Additionally, 1 µL of PDS (10 µm) or 1 µL of Milli‐Q water (serving as the control) was added, along with varying concentrations of the EBV ds‐target, with Milli‐Q water added to achieve a final volume of 20 µL. The resulting mixture was incubated at 37 °C in a Celemetor‐96 for 60 min, during which fluorescence readings were recorded every 30 s.

### Inhibition Assay for CRISPR‐Cas12a's *trans‐*Cleavage Activity Using G4‐DR

Pre‐incubated 2 µL of DR or G4‐DR (400 nm, Table S1, Supporting Information) at room temperature in conjunction with a concentration gradient of PDS and KCl. Milli‐Q water was added to reach a final volume of 10 µL within a 0.2 mL microcentrifuge tube, and the mixture was incubated for 10 min. Subsequently, 2 µL of the 10× LbCas12a Reaction Buffer, 1 µL of the chosen Spacer (either EBV spacer, HCV spacer, or MPXV spacer, 800 nm, Table S1, Supporting Information), 1 µL of the corresponding ds target (EBV target, HCV target, or MPXV target, 80 nm), 1 µL of the F‐Q reporter (5 µm), and 2 µL of LbCas12a (1 µm) were introduced. The total volume was adjusted to 20 µL with Milli‐Q water. The resulting mixtures were incubated at 37 °C for 20 min in a Celemetor‐96, with fluorescence readings recorded every 20 s.

### Determining the Cleavage Time of the PC Linker in G4‐PC‐DR

To determine the time required for the breakage of the PC linker in G4‐PC‐DR under ultraviolet (UV) light exposure (λ = 365 nm, 35 W), a mixture was prepared which containing a final concentration of 1x LbCas12a reaction buffer, 1 µm G4‐PC‐DR and nuclease‐free water to achieve a final volume of 10 µL. Subsequently, the mixture was subjected to UV light exposure for 0, 2, 4, 8, 16, 32, and 60 s. To evaluate the breakage of the PC linker after UV light exposure, the samples were analyzed using denaturing 20% PAGE with 7 m urea after DNA dye staining.

### Photo‐Controlled CRISPR‐Cas12a System of *cis‐* and *trans‐*Cleavage Activity Using G4‐PC‐DR

Similar to the inhibition assay, 2 µL of either DR or G4‐DR (400 nm, Table S1, Supporting Information) was pre‐incubated at room temperature with 2 µL of PDS (10 µm), 1 µL of KCl (500 mm), and 5 µL of Milli‐Q water for 10 min. To investigate the regulation by UV light, two experimental groups were established: one exposed to UV light and a control group that was not, with an exposure duration of 30 s.

To assess the *cis*‐cleavage activity of the photo‐controlled CRISPR‐Cas12a system, the following components were subsequently added: 2 µL of the 10× LbCas12a Reaction Buffer, 1 µL of the EBV Spacer (800 nm, Table S1, Supporting Information), 1 µL of FAM‐labeled ds EBV target (800 nm), and 2 µL of LbCas12a (1 µm). The total volume of the reaction was then adjusted to 20 µL with Milli‐Q water. The mixtures were incubated at 37 °C for 30 min in a Celemetor‐96. Following this, 40 µg of Proteinase K was added, and the reaction was maintained at 37 °C for an additional 15 min to facilitate protein digestion. The cis‐cleavage activity of LbCas12a was subsequently analyzed using 2% agarose gel electrophoresis (120 V for ≈30 min).

To validate the *trans*‐cleavage activity of the photo‐controlled CRISPR‐Cas12a system, a similar pre‐incubation and UV exposure procedure was applied. Following this, the components added to the reaction included 2 µL of the 10× LbCas12a Reaction Buffer, 1 µL of the EBV Spacer (800 nm, Table S1, Supporting Information), 1 µL of ds EBV target (20 nm), 2 µL of either LbCas12a, AsCas12a, ErCas12a, or FnCas12a (1 µM), and 1 µL of the F‐Q reporter (5 µm). The total reaction volume was adjusted to 20 µL using Milli‐Q water. The mixtures were incubated at 37 °C for 20 min in a Celemetor‐96, with fluorescence readings recorded every 20 s.

### Classical One‐Pot RPA‐CRISPR Cas12a System for DNA Detection Using crRNA

Initially, each lyophilized RPA pellet was reconstituted in 25 µL of Milli‐Q water while being kept on ice. For each reaction, 10 µL of the RPA mixture was carefully transferred to a pre‐cooled 0.2 mL microcentrifuge tube. To this, the following components were added: 1 µL of RPA forward primer (2 µm), 1 µL of RPA reverse primer (2 µm), 1 µL of classical crRNA (800 nm), 1 µL of the F‐Q reporter (5 µm), 1 µL of LbCas12a (2 µm), and 2 µL of the 10× RPA‐Cas12a buffer. Additionally, 2 µL of a standard ds target with varying copy numbers or a clinical sample was included, with Milli‐Q water added to reach a final volume of 20 µL. The resulting mixtures were incubated at 37 °C in a Celemetor‐96 for 45 min, with fluorescence readings recorded every 30 s.

### Photo‐Controlled One‐Pot RPA‐Cas12a System for DNA Detection Using G4‐PC‐DR

Each lyophilized RPA pellet was first reconstituted in 25 µL of Milli‐Q water while maintaining a temperature on ice. For each individual reaction, 10 µL of the RPA mixture was transferred into a pre‐cooled 0.2 mL microcentrifuge tube, supplemented with the following components: 1 µL of RPA forward primer (2 µm), 1 µL of RPA reverse primer (2 µm), 1 µL of G4‐PC‐DR (800 nm), 1 µL of the target corresponding Spacer (800 nm), 1 µL of the F‐Q reporter (5 µm), 1 µL of LbCas12a (2 µm), and 2 µL of the 10× RPA‐Cas12a buffer. Additionally, 2 µL of the standard ds target with varying copy numbers or a clinical sample was included to achieve a final volume of 20 µL. The resulting mixtures were incubated at 37 °C in a Celemetor‐96 for 15 min, with fluorescence readings taken every 5 min. Following amplification, the mixture tubes were removed and exposed to UV light (λ = 365 nm, 35 W) for 30 s, after which they were incubated at 37 °C in the Celemetor‐96 for an additional 60 min. Fluorescent readings were collected every minute for a total of 60 min. The DNA content in the clinical samples was determined based on the fluorescent data generated from the standard values.

### Real‐Time Quantitative PCR (qPCR) for Clinical EBV Sample Detection

A traditional qPCR was performed to validate the accuracy of the photo‐controlled one‐pot method for detecting clinical EBV samples. Each reaction contained 0.2 µm of each primer (EBV PCR‐F and EBV PCR‐R, Table S1, Supporting Information) and 2 µL of either a template with different concentrations (10^2^–10^7^ copies mL^−1^) of the EBV target for generating a standard curve or clinical samples. The total reaction volume was adjusted to 20 µL with Hieff 1x qPCR SYBR Green Master Mix (No Rox) and DNase/RNase‐free water. The mixture was then subjected to a PCR run on Celemetor‐96, with an initial denaturation at 95 °C for 3 min, followed by 40 cycles of amplification at 95 °C for 10 s and 55 °C for 30 s. The EBV content in clinical samples was quantified based on the Ct values and standard curve.

### Statistical Analysis

All data are presented as mean ± standard deviation (SD). Student's *t*‐test or one‐way analysis of variance (ANOVA) was used to assess statistical significance. A *p*‐value less than 0.05 was considered statistically significant.

## Conflict of Interest

The authors declare no conflict of interest.

## Supporting information



Supporting Information

## Data Availability

The data that support the findings of this study are available in the supplementary material of this article.
