# Supplementary material for: Expanding Cas12a Activity Control with an RNA G‐Quadruplex at the 5′ end of CRISPR RNA
Source: Adv Sci (Weinh). 2024 Dec 25;12(7):2411305. doi: 10.1002/advs.202411305 (PMC11831528; doi:10.1002/advs.202411305)
Supplement: Supplementary file 1 — Supporting Information [file ADVS-12-2411305-s001.pdf]

ADVANCED SCIENCE
Open Access

Supporting Information

for *Adv. Sci.*, DOI 10.1002/advs.202411305

Expanding Cas12a Activity Control with an RNA G-Quadruplex at the 5′ end of CRISPR RNA

Wenjuan Huang, Jiaqi Wang, Cheng Wang, Yuanfang Liu, Wentao Li, Qiaozhen Chen, Junqiu Zhai, Zhenyang Xiang* and Chaoxing Liu**

Supplementary Information

Expanding Cas12a Activity Control with an RNA G-quadruplex at the 5' End of CRISPR RNA

Wenjuan Huang,[1†] Jiaqi Wang,[2†] Cheng Wang,[1†] Yuanfang Liu,[2] Wentao Li,[3] Qiaozhen Chen,[4] Junqiu Zhai,[5] Zhenyang Xiang,[1*] and Chaoxing Liu[2*]*

[1] Taizhou Hospital of Zhejiang Province Affiliated to Wenzhou Medical University, Linhai, 317000, P. R. China.

[2] Guangdong Provincial Key Laboratory of Digestive Cancer Research, Digestive Diseases Center, Scientific Research Center, The Seventh Affiliated Hospital of Sun Yat-sen University, Shenzhen, 518107, P. R. China.

[3] Medical Laboratory Department, The Seventh Affiliated Hospital of Sun Yat-sen University, Shenzhen, 518107, P. R. China.

[4] School of Chemistry and Biological Engineering, University of Science and Technology Beijing, Beijing, 100083, P. R. China

[5] School of Pharmaceutical Sciences, Guangzhou University of Chinese Medicine, Guangzhou, 510006, P. R. China.

[†] These authors contributed equally.

* Corresponding Author: Junqiu Zhai, Email: jqzhai@gzucm.edu.cn; Zhenyang Xiang, Email: xiangzy@enzemed.com; Chaoxing Liu, Email: liuchx69@mail.sysu.edu.cn

S1. ODN sequences used in this study

Table S1. Sequences of DNA or RNA oligonucleotides used in this work.

Name	Sequence (from 5' to 3')	Notes
RS crRNA	AAUUUCUACUAAGUGUAGAUUUAUCGCAACUUUCUACUGAAUU	RNA
RS G4-crRNA (telo-crRNA)	GGGUUAGGGUUAGGGUUAGGGAAUUUCUACUAAGUGUAGAUUUAUCGCAACUUUCUACUGAAUU	RNA, red part is Telo-RG4 sequences
KRAS-crRNA	GGGCGGUGUGGGAAUAGGGAAUUUCUACUAAGUGUAGAUUUAUCGCAACUUUCUACUGAAUU	RNA, red part is KRAS-RG4 sequences
VEGF-crRNA	GGGCGGGCCGGGGGCGGGAAUUUCUACUAAGUGUAGAUUUAUCGCAACUUUCUACUGAAUU	RNA, red part is VEGF-RG4 sequences
ckit-crRNA	GGGAGGGCGCUGGGAGGUGGGAAUUUCUACUAAGUGUAGAUUUAUCGCAACUUUCUACUGAAUU	RNA, red part is ckit-RG4 sequences
G4-crRNA-FAM	5`6-FAM-GGGUUAGGGUUAGGGUUAGGGAAUUUCUACUAAGUGUAGAUUUAUCGCAACUUUCUACUGAAUU	RNA, 5`6-FAM modification, red part is Telo-RG4 sequences
G4mut-crRNA	G**A**GUUAG**A**GUUAG**A**GUUAG**A**GAAUUUCUACUAAGUGUAGAUUUAUCGCAACUUUCUACUGAAUU	RNA, black bold parts are four Gs mutation of Telo-RG4 sequences
G4mut-crRNA-FAM	5`6-FAM-G**A**GUUAG**A**GUUAG**A**GUUAG**A**GAAUUUCUACUAAGUGUAGAUUUAUCGCAACUUUCUACUGAAUU	RNA, 5`6-FAM modification, black bold parts are four Gs mutation of Telo-RG4 sequences
RS target	CCGAATTCAGTAGAAAGTTGCGATAACAAAACTGGCCGTCGTTTTACAAC	DNA
RS target-CS	GTTGTAAAACGACGGCCAGTTTTGTTATCGCAACTTTCTACTGAATTCGG	DNA, RS target complementary sequences
Telo G4	GGGTTAGGGTTAGGGTTAGGG	DNA, red part is DNA Telo-G4 sequences
Telo G4-CS	CCCTAACCCTAACCCTAACCC	DNA, Telo G4 complementary sequences
Telo G4 mut	GAGTTAGAGTTAGAGTTAGAG	DNA
EBV crRNA	AAUUUCUACUAAGUGUAGAUCAAACUCAUAUAUUUGCUGA	RNA
G4-EBV crRNA	GGGUUAGGGUUAGGGUUAGGGAAUUUCUACUAAGUGUAGAUCAAACUCAUAUAUUUGCUGA	RNA, red part is Telo-RG4 sequences
G4mut-EBV crRNA	G**A**GUUAG**A**GUUAG**A**GUUAG**A**GAAUUUCUACUAAGUGUAGAUCAAACUCAUAUAUUUGCUGA	RNA, black bold parts are four Gs mutation of Telo-RG4 sequences
DR	AAUUUCUACUAAGUGUAGAU	RNA
G4-DR	GGGUUAGGGUUAGGGUUAGGGAAUUUCUACUAAGUGUAGAU	RNA, red part is Telo-RG4 sequences
G4-DR-FAM	5`6-FAM-GGGUUAGGGUUAGGGUUAGGGAAUUUCUACUAAGUGUAGAU	RNA, 5`6-FAM modification, red part is Telo-RG4 sequences
G4mut-DR	G**A**GUUAG**A**GUUAG**A**GUUAG**A**GAAUUUCUACUAAGUGUAGAU	RNA, black bold parts are four Gs mutation of Telo-RG4 sequences
G4mut-DR-FAM	5`6-FAM-G**A**GUUAG**A**GUUAG**A**GUUAG**A**GAAUUUCUACUAAGUGUAGAU	RNA, 5`6-FAM modification, black bold parts are four Gs mutation of Telo-RG4 sequences

G4-PC-DR	GGGUUAGGGUUAGGGUUAGGG/PC Linker/AAUUUCUACUAAGUGUAGAU	RNA, red part is Telo-RG4 sequences
EBV Spacer	CAAACUCAUAUAUUUGCUGA	RNA
HCV Spacer	GGCGUGCCCCCGCAAGACUG	RNA
MPXV Spacer	GUAUAUAAGUUGUACGGCUAAUUC	RNA
EBV RPA-F	GACCCGGCCCACAACCTGGCCCACTAAGGG	DNA, RPA forward primer for EBV target
EBV RPA-imotif-F (iF)	CCCTAACCCTAACCCTAACCCGACCCGGCCCACAACCTGGCCCACTAAGGG	DNA, RPA forward primer for G-rich EBV target
EBV RPA-R	ACTCCATCGTCAAAGCTGCACACAGTCACC	DNA, RPA reverse primer for EBV target
EBV PCR-F	AATACCTCCAAGAAGGTGGC	DNA, PCR forward primer for EBV target
EBV PCR-R	TGCTCACCAGGTAAATGTCG	DNA, PCR reverse primer for EBV target
EBV PCR-R-FAM	5`6-FAM-TGCTCACCAGGTAAATGTCG	DNA, FAM labeled reverse primer for EBV target
HCV PCR-F	GTCTTCACGCAGAAAGCGTC	DNA, PCR forward primer for HCV target
HCV PCR-R	AGGATTCGTGCTCATGGTGC	DNA, PCR reverse primer for HCV target
MPXV PCR-F	GTAAAACGACGGCCAGT	DNA, PCR forward primer for MPXV target
MPXV PCR-R	CAGGAAACAGCTATGAC	DNA, PCR reverse primer for MPXV target
FAM-Biotin reporter	5`6-FAM-CCCCCCCC-3`Biotin	DNA, 5`6-FAM and 3`Biotin modifications
F-Q reporter	5`6-FAM-CCCCCCCC-3`BHQ1	DNA, 5`6-FAM and 3`BHQ1 modifications

Abbreviations and modifications:

/PC Linker/: It links two nucleotide sequences via a short, UV-photocleavable C3 spacer arm that can be attached at any position in the sequence.

/56-FAM/: 5' 6-FAM (Fluorescein) modification

/BHQ1/: Black Hole Quencher-1, which is used to quench green and yellow dyes, such as FAM, TET, and HEX.

Table S2. Sequences of plasmids used in this work.

Name	Sequence (5‘→3‘)
T7-HCV IRES	TAATACGACTCACTATAGGGAACTACTGTCTTCACGCAGAAAGCGTCTAGCCATGGCGTTAGT<br>ATGAGTGTCGTGCAGCCTCCAGGACCCCCCCTCCCGGGAGAGCCATAGTGGTCTGCGGAAC<br>CGGTGAGTACACCGGAATTGCCAGGACGACCGGGTCCTTTCTTGGATAAACCCGCTCAATGC<br>CTGGAGATTTGGGCGTGCCCCCGCAAGACTGCTAGCCGAGTAGTGTTGGGTCGCGAAAGGC<br>CTTGTGGTACTGCCTGATAGGGTGCTTGCGAGTGCCCCGGGAGGTCTCGTAGACCGTGCACC<br>ATGAGCACGAATCCTAAACCTCAAAGA
MPXV B6R	ATGAAAACGATTTCCGTTGTTACGTTGTTATGCGTACTACCTGCTGTTGTTTATTCAACATGTA<br>CTGTACCCACTATGAATAACGCTAAATTAACGTCTACCGAAACATCGTTTAATGATAAACAGA<br>AAGTTACGTTTACATGTGATTCAGGATATCATTCTTTGGATCCAAATGCTGTCTGTGAAACAG<br>ATAAATGGAAATACGAAAATCCATGCAAGAAAATGTGCACAGTTTCTGATTATGTCTCTGAAC<br>TATATGATAAGCCATTATACGAAGTGAATTCCACCATGACACTAAGTTGCAACGGTGAAACAA<br>AATATTTTCGTTGTGAAGAAAAAAATGGAAATACTTCTTGGAATGATACTGTCACGTGTCCTA<br>ATGCGGAATGTCAACCTCTTCAATTAGAACACGGATCGTGTCAACCAGTTAAAGAAAAATAC<br>TCATTTGGGGAATATATGACTATCAACTGTGATGTTGGATATGAGGTTATTGGTGTTTCGTATAT<br>AAGTTGTACGGCTAATTCTTGGAATGTTATTCCATCATGTCAACAAAAATGTGATATACCGTCC<br>CTATCTAATGGATTAATTTCCGGATCTACATTTTCTATCGGTGGCGTTATACATCTTAGTTGTAA<br>AAGTGGTTTTACACTAACGGGGTCTCCATCATCCACATGTATCGACGGTAAATGGAATCCCAT<br>ACTCCCAACATGTGTACGATCTAACGAAGAATTTGATCCAGTGGATGATGGTCCCGACGATG<br>AGACAGATCTGAGCAAACTCTCGAAAGACGTTGTACAATATGAACAAGAAATAGAATCGTTA<br>GAAGCAACTTATCATATAATCATAATGGCGTTGACAATTATGGGTGTCATATTTCTAATCTCCAT<br>TATAGTATTAGTTTGTTCCTGTGACAAAAATAATGACCAATATAAGTTCCATAAATTGCTACCG<br>TGA
pUC57<br>(Notes: Cloning vector, cloning sites: XbaI and SmaI)	TCGCGCGTTTCGGTGATGACGGTGAAAACCTCTGACACATGCAGCTCCCGGAGACGGTCAC<br>AGCTTGTCTGTAAGCGGATGCCGGGAGCAGACAAGCCCGTCAGGGCGCGTCAGCGGGTGTT<br>GGCGGGTGTCGGGGCTGGCTTAACTATGCGGCATCAGAGCAGATTGTACTGAGAGTGCACCA<br>TATGCGGTGTGAAATACCGCACAGATGCGTAAGGAGAAAATACCGCATCAGGCGCCATTCGC<br>CATTCAGGCTGCGCAACTGTTGGGAAGGGCGATCGGTGCGGGCCTCTTCGCTATTACGCCAG<br>CTGGCGAAAGGGGGATGTGCTGCAAGGCGATTAAGTTGGGTAACGCCAGGGTTTTCCCAGT<br>CACGACGTTGTAAAACGACGGCCAGTGAATTCGAGCTCGGTACCTCGCGAATGCATCTAGAT<br>ATCGGATCCCGGGCCCGTCGACTGCAGAGGCCTGCATGCAAGCTTGGCGTAATCATGGTCAT<br>AGCTGTTTCCTGTGTGAAATTGTTATCCGCTCACAATTCCACACAACATACGAGCCGGAAGC<br>ATAAAGTGTAAAGCCTGGGGTGCCTAATGAGTGAGCTAACTCACATTAATTGCGTTGCGCTCA<br>CTGCCCGCTTTCCAGTCGGGAAACCTGTCGTGCCAGCTGCATTAATGAATCGGCCAACGCGC<br>GGGGAGAGGCGGTTTGCGTATTGGGCGCTCTTCCGCTTCCTCGCTCACTGACTCGCTGCGCT<br>CGGTCGTTCGGCTGCGGCGAGCGGTATCAGCTCACTCAAAGGCGGTAATACGGTTATCCACA<br>GAATCAGGGGATAACGCAGGAAAGAACATGTGAGCAAAAGGCCAGCAAAAGGCCAGGAAC<br>CGTAAAAAGGCCGCGTTGCTGGCGTTTTTCCATAGGCTCCGCCCCCCTGACGAGCATCACAA<br>AAATCGACGCTCAAGTCAGAGGTGGCGAAACCCGACAGGACTATAAAGATACCAGGCGTTT

	CCCCCTGGAAGCTCCCTCGTGCGCTCTCCTGTTCCGACCCTGCCGCTTACCGGATACCTGTCC<br>GCCTTTCTCCCTTCGGGAAGCGTGGCGCTTTCTCATAGCTCACGCTGTAGGTATCTCAGTTCG<br>GTGTAGGTCGTTCGCTCCAAGCTGGGCTGTGTGCACGAACCCCCCGTTCAGCCCGACCGCTG<br>CGCCTTATCCGGTAACTATCGTCTTGAGTCCAACCCGGTAAGACACGACTTATCGCCACTGGC<br>AGCAGCCACTGGTAACAGGATTAGCAGAGCGAGGTATGTAGGCGGTGCTACAGAGTTCTTGA<br>AGTGGTGGCCTAACTACGGCTACACTAGAAGAACAGTATTTGGTATCTGCGCTCTGCTGAAG<br>CCAGTTACCTTCGGAAAAAGAGTTGGTAGCTCTTGATCCGGCAAACAAACCACCGCTGGTAG<br>CGGTGGTTTTTTTGTTTGCAAGCAGCAGATTACGCGCAGAAAAAAAGGATCTCAAGAAGATC<br>CTTTGATCTTTTCTACGGGGTCTGACGCTCAGTGGAACGAAAACTCACGTTAAGGGATTTTG<br>GTCATGAGATTATCAAAAAGGATCTTCACCTAGATCCTTTTAAATTAAAAATGAAGTTTTAAAT<br>CAATCTAAAGTATATATGAGTAAACTTGGTCTGACAGTTACCAATGCTTAATCAGTGAGGCAC<br>CTATCTCAGCGATCTGTCTATTTCGTTCATCCATAGTTGCCTGACTCCCCGTCGTGTAGATAAC<br>TACGATACGGGAGGGCTTACCATCTGGCCCCAGTGCTGCAATGATACCGCGAGgCCCACGCT<br>CACCGGCTCCAGATTTATCAGCAATAAACCAGCCAGCCGGAAGGGCCGAGCGCAGAAGTGG<br>TCCTGCAACTTTATCCGCCTCCATCCAGTCTATTAATTGTTGCCGGGAAGCTAGAGTAAGTAG<br>TTCGCCAGTTAATAGTTTGCGCAACGTTGTTGCCATTGCTACAGGCATCGTGGTGTCACGCTC<br>GTCGTTTGGTATGGCTTCATTCAGCTCCGGTTCCCAACGATCAAGGCGAGTTACATGATCCCC<br>CATGTTGTGCAAAAAAGCGGTTAGCTCCTTCGGTCCTCCGATCGTTGTCAGAAGTAAGTTGG<br>CCGCAGTGTTATCACTCATGGTTATGGCAGCACTGCATAATTCTCTTACTGTCATGCCATCCGT<br>AAGATGCTTTTCTGTGACTGGTGAGTACTCAACCAAGTCATTCTGAGAATAGTGTATGCGGCG<br>ACCGAGTTGCTCTTGCCCGGCGTCAATACGGGATAATACCGCGCCACATAGCAGAACTTTAA<br>AAGTGCTCATCATTGGAAAACGTTCTTCGGGGCGAAAACTCTCAAGGATCTTACCGCTGTTG<br>AGATCCAGTTCGATGTAACCCACTCGTGCACCCAACTGATCTTCAGCATCTTTTACTTTCACC<br>AGCGTTTCTGGGTGAGCAAAAACAGGAAGGCAAAATGCCGCAAAAAAGGGAATAAGGGCG<br>ACACGGAAATGTTGAATACTCATACTCTTCCTTTTTCAATATTATTGAAGCATTTATCAGGGTT<br>ATTGTCTCATGAGCGGATACATATTTGAATGTATTTAGAAAAATAAACAAATAGGGGTTCCGC<br>GCACATTTCCCCGAAAAGTGCCACCTGACGTCTAAGAAACCATTATTATCATGACATTAACCT<br>ATAAAAATAGGCGTATCACGAGGCCCTTTCGTC

S2. Preparation of ds DNA Target

For RS target: To obtain the RS ds target, RS target (10 μM, Table S1) and RS target-CS (10 μM, Table S1) were incubated at 95°C in a final 1x LbCas12a reaction buffer (0.01% tween 20, 40 mM Glycine, 10 mM Tris-HCl, 1 mM DTT, 40 mM KCl, 32.4 mM $MgCl_2$, pH 8.5) for 5 min, then annealed to 4°C and stored at -20°C.

For Epstein-Barr Virus (EBV) target: A mixture containing 0.1 μM of each primer (EBV PCR-F, EBV PCR-R or EBV PCR-R-FAM, Table S1), 1x Canace® Plus PCR buffer, and 1 unit of Hieff Canace® Plus High-Fidelity DNA Polymerase was prepared. Additionally, 2 μL of EBV clinical sample extract (collected from the Laboratory Department of the Seventh Affiliated Hospital of Sun Yat-sen University, with a concentration of $9x10^4$ copies/mL) was included in a final volume of 50 μL using DNase/RNase-free water. The PCR was performed according to routine PCR protocol. The final PCR products were purified using the MolPure® PCR Purification Kit.

For Hepatitis C Virus (HCV) target: The HCV plasmid (Table S2) carrying the HCV fragment was linearized using restriction enzymes and transcribed with the HiScribe® T7 High Yield RNA Synthesis Kit following the manufacturer's instructions. The HCV target was purified using RNA clean beads and stored at -20°C.

For Monkeypox Virus (MPXV) target: The MPXV plasmid (Table S2) carrying the MPXV fragment was used as templates. All other conditions remained consistent with those described for the EBV target except the primers were changed to MPXV primer (Table S1).

S3. Extended Data

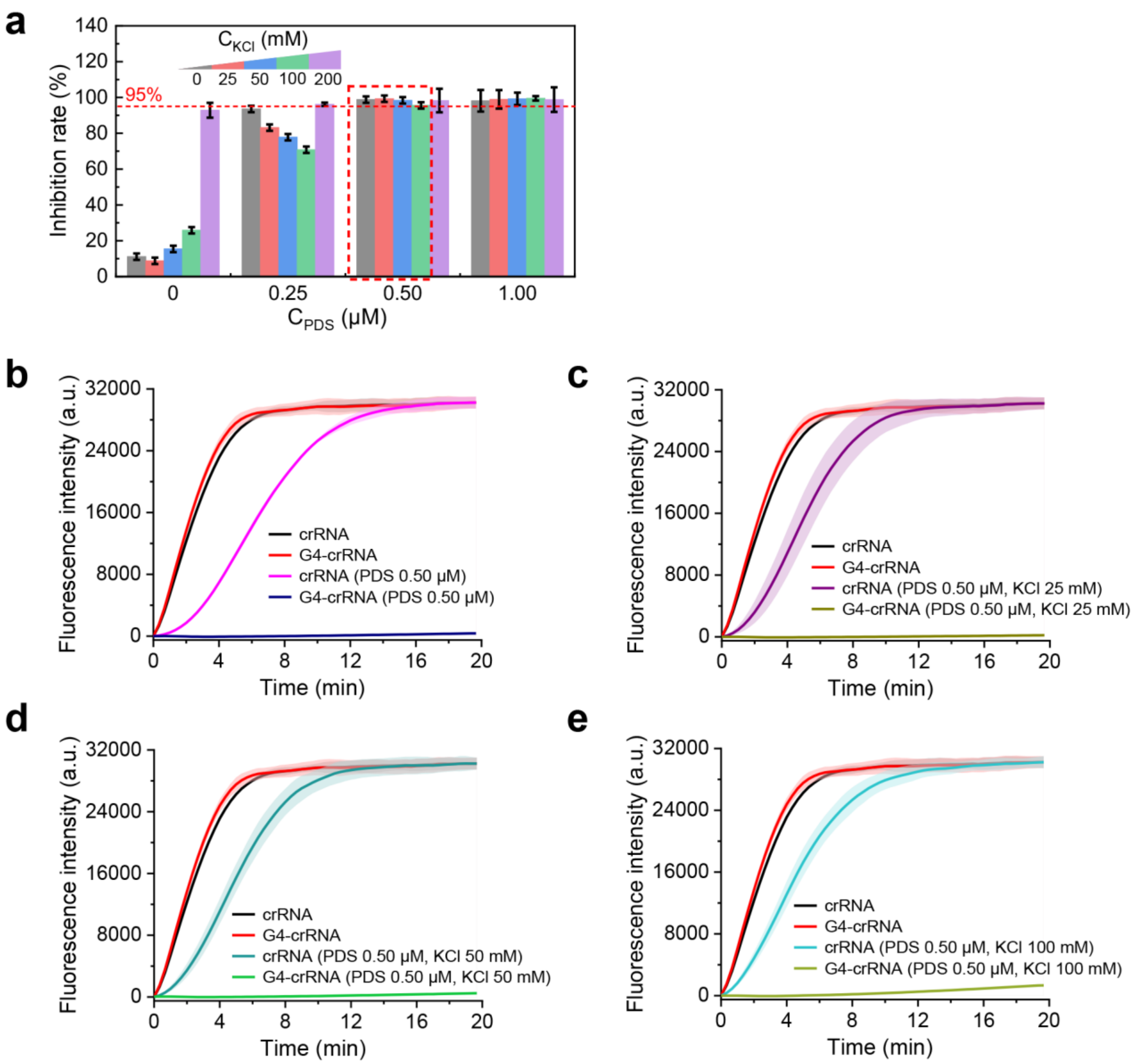

Figure S1. (a) Bar graph of the cleavage activity inhibition rate of G4-crRNA assisted CRISPR-Cas12a system under different concentrations of KCl and PDS. At PDS 0.5 μM and KCl ranging from 0 to 100 mM, the inhibition rate exceeds 95%. Error bars are standard deviation (SD) (n = 3). (b-e) Real-time fluorescence graphs depicting the activity of classic crRNA and G4-crRNA assisted CRISPR-Cas12a system at PDS 0.5 μM with KCl concentrations of 0 mM (b), 25 mM (c), 50 mM (d), and 100 mM (e). Error bars are standard deviation (SD) (n = 3).

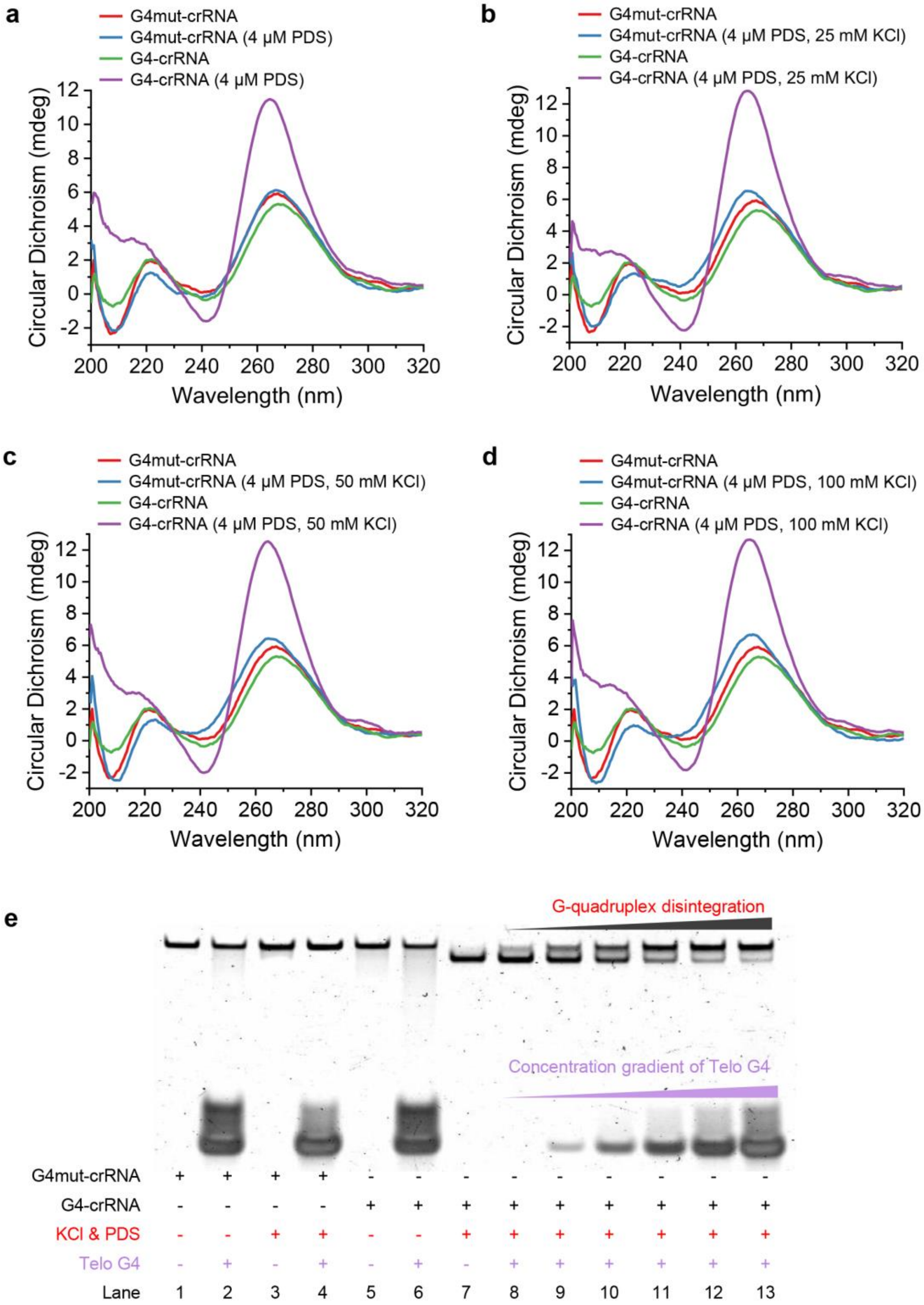

Figure S2. Circular dichroism (CD) spectra of 8 μM G4mut-crRNA or G4-crRNA in the presence of 4 μM PDS, under varying concentrations of KCl: 0 mM (a), 25 mM (b), 50 mM (c), and 100 mM (d). The purple

line indicates that PDS effectively promotes the formation of G4 structures with G4-crRNA across all KCl concentrations tested (0 to 100 mM), while G4mut-crRNA does not exhibit this capability. (e) Native PAGE was utilized to visualize the secondary structure alterations of G4mut-crRNA and G4-crRNA following the introduction of varying concentrations of ss-Telo G4 under conditions of PDS 0.5 μM and 50 mM KCl. This analysis revealed that the escalating levels of ss-Telo G4 compete with the G4 stabilizers, displacing the stable RG4 structure inherent in G4-crRNA. Consequently, this competition prompts a reversion of the RG4 structure to a configuration reminiscent of G4mut-crRNA.

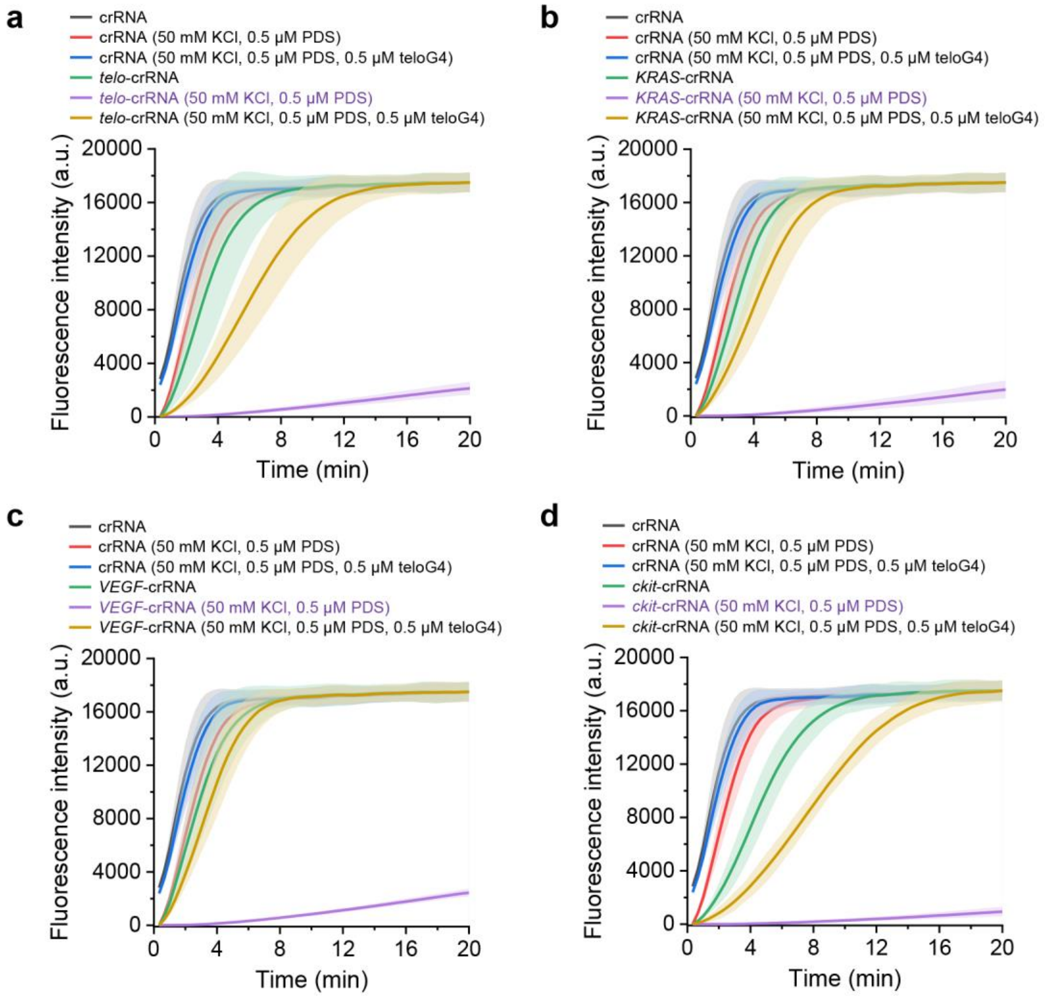

Figure S3. Real-time fluorescence comparison of crRNAs with different 5' end RG4 structures: telomere (a), KRAS (b), VEGF (c), and c-Kit (d), under conditions of 50 mM KCl and 0.5 μM PDS. Additionally, the comparison includes conditions with an extra 0.5 μM DNA telomeric sequence. These conditions are benchmarked against classical crRNA under identical conditions. Error bars are standard deviation (SD) (n = 3).

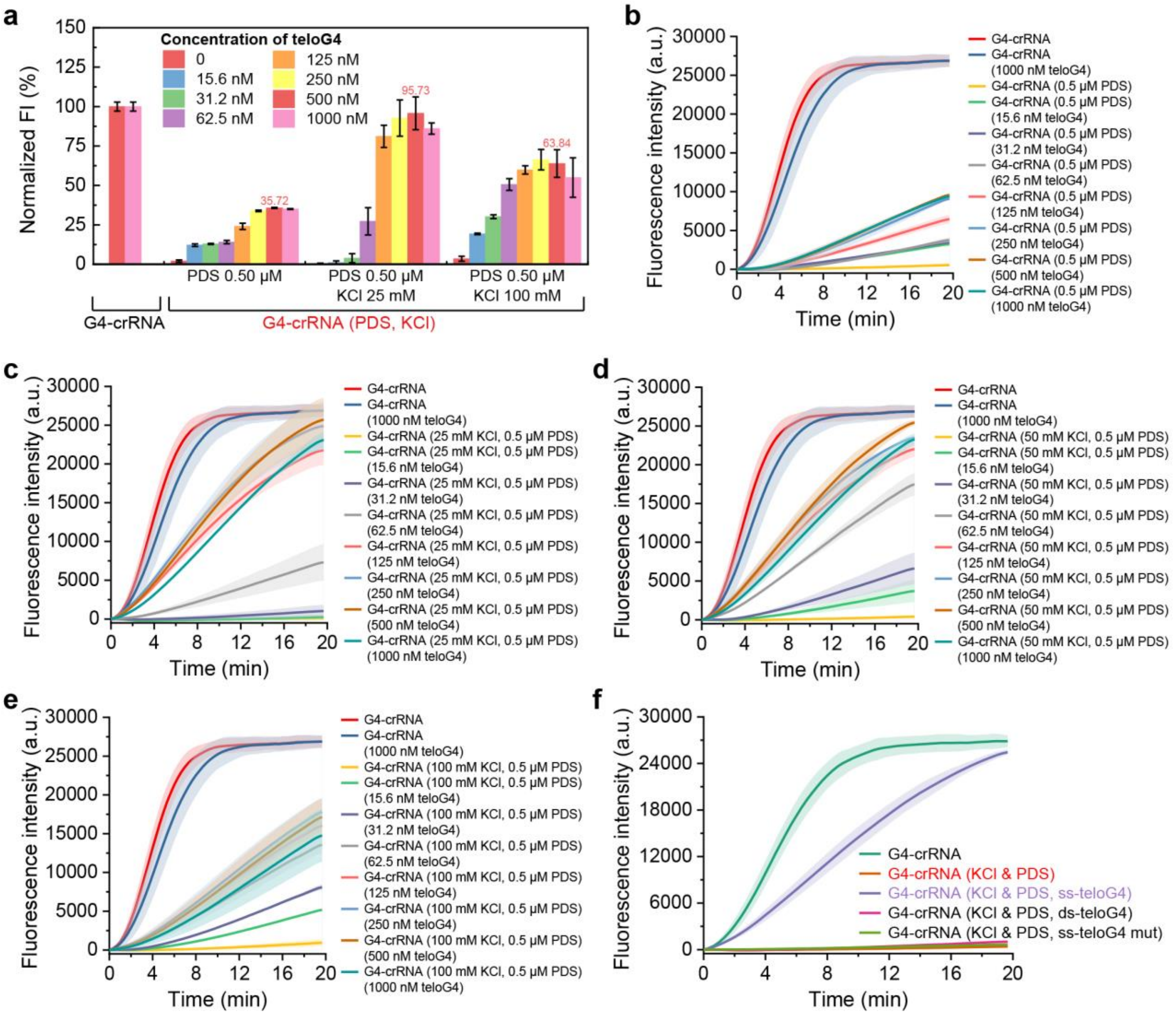

Figure S4. (a) A bar graph illustrating the inhibition and subsequent recovery of G4-crRNA *trans*-cleavage activity in the presence of 0.5 μM PDS plus 0, 25 or 100 mM KCl, as the concentration of exogenous Telo G4 increases. Error bars are standard deviation (SD) (n = 3). (b-e) A real-time fluorescence plot demonstrating the reactivation of the CRISPR-Cas12a system in the presence of varying concentrations of single-stranded Telo G4, with 0.5 μM PDS plus 0 (b), 25 (c), 50 (d), or 100 mM KCl (e). Error bars are standard deviation (SD) (n = 3). (f) Real-time fluorescence graph illustrating the recovery of Cas12a cleavage activity in the presence of 500 nM ss-Telo G4, ds-Telo G4, or a ss-Telo G4 mutant (with four mutated guanines that prevent the formation of the G4 structure), under PDS 0.5 μM and 50 mM KCl. Error bars are standard deviation (SD) (n = 3).

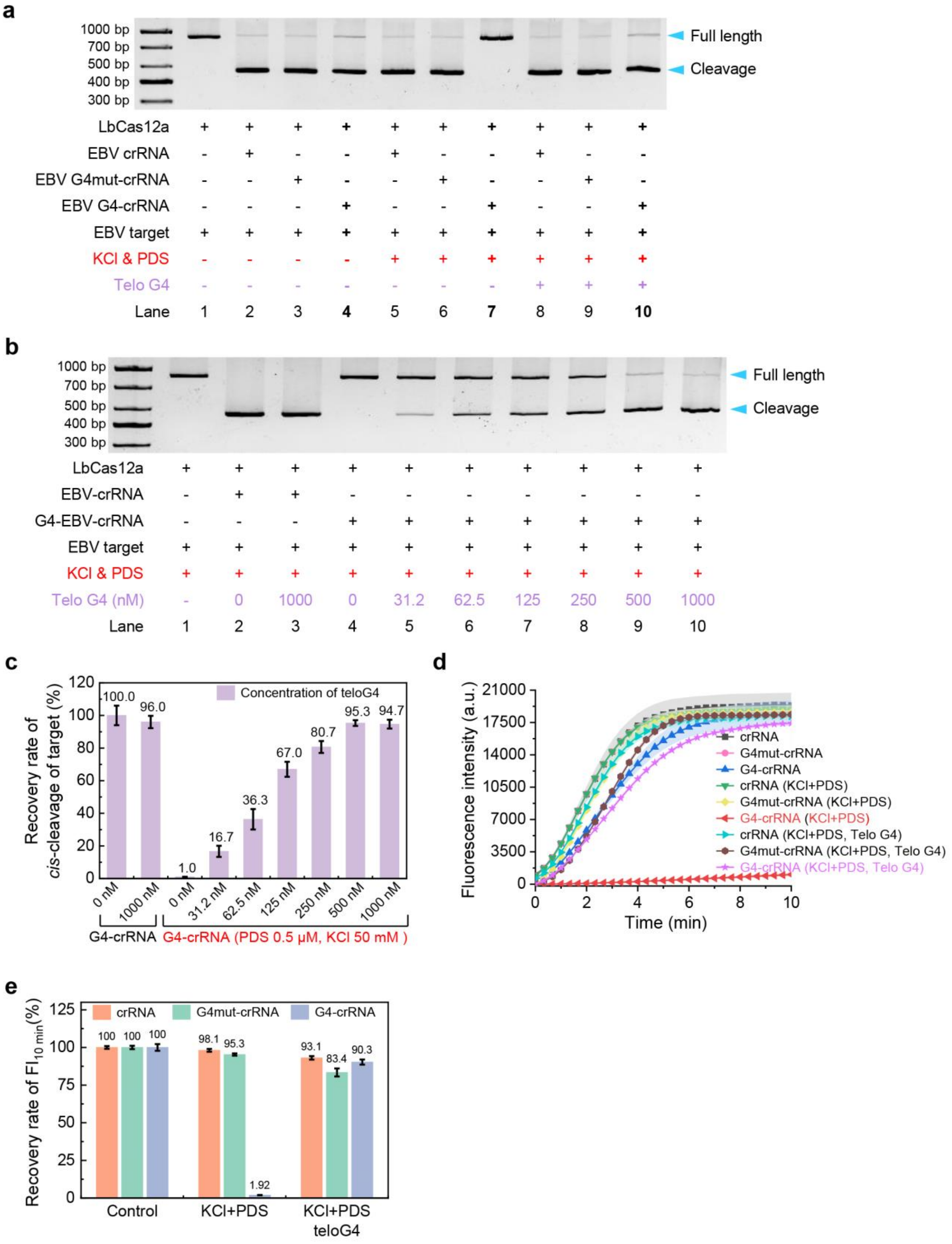
a
1000 bp
700 bp
500 bp
400 bp
300 bp
Full length
Cleavage
LbCas12a + + + + + + + + + +
EBV crRNA - + - - + - - + - -
EBV G4mut-crRNA - - + - - + - - + -
EBV G4-crRNA - - - + - - + - - +
EBV target + + + + + + + + + +
KCl & PDS - - - - + + + + + +
Telo G4 - - - - - - - + + +
Lane 1 2 3 4 5 6 7 8 9 10
b
1000 bp
700 bp
500 bp
400 bp
300 bp
Full length
Cleavage
LbCas12a + + + + + + + + + +
EBV-crRNA - + + - - - - - - -
G4-EBV-crRNA - - - + + + + + + +
EBV target + + + + + + + + + +
KCl & PDS + + + + + + + + + +
Telo G4 (nM) - 0 1000 0 31.2 62.5 125 250 500 1000
Lane 1 2 3 4 5 6 7 8 9 10
c
Recovery rate of cis-cleavage of target (%)
Concentration of teloG4
100.0
96.0
1.0
16.7
36.3
67.0
80.7
95.3
94.7
0 nM
1000 nM
0 nM
31.2 nM
62.5 nM
125 nM
250 nM
500 nM
1000 nM
G4-crRNA
G4-crRNA (PDS 0.5 μM, KCl 50 mM)
d
Fluorescence intensity (a.u.)
Time (min)
crRNA
G4mut-crRNA
G4-crRNA
crRNA (KCl+PDS)
G4mut-crRNA (KCl+PDS)
G4-crRNA (KCl+PDS)
crRNA (KCl+PDS, Telo G4)
G4mut-crRNA (KCl+PDS, Telo G4)
G4-crRNA (KCl+PDS, Telo G4)
e
Recovery rate of FI10 min(%)
crRNA
G4mut-crRNA
G4-crRNA
100
100
100
98.1
95.3
1.92
93.1
83.4
90.3
Control
KCl+PDS
KCl+PDS teloG4

Figure S5. (a) Agarose gel image of the cis-cleavage activity of Cas12a targeting the EBV target with classic EBV crRNA, EBV G4mut-crRNA, or EBV G4-crRNA, under conditions with or without G4 stabilizers and Telo G4. The results demonstrate that only G4-crRNA with G4 stabilizers (KCl & PDS) inhibits Cas12a activity (Lane 7), and this inhibition is reversed by the addition of Telo G4 (Lane 10). (b) Agarose gel image showing the gradual restoration of *cis*-cleavage activity of Cas12a targeting the EBV target with classic EBV G4-crRNA, under increasing concentrations of Telo G4 in the presence of G4 stabilizers. (c) Quantitative bar graph from figure b. Error bars are standard deviation (SD) (n = 3). (d) Real-time fluorescence graph illustrating the *trans*-cleavage activity towards a single-stranded fluorescent reporter FQ in the presence of target EBV, for Cas12a with classic EBV crRNA, EBV G4mut-crRNA, or EBV G4-crRNA, under conditions with and without G4 stabilizers and Telo G4. The results show that only G4-crRNA with G4 stabilizers (KCl & PDS) inhibits *trans*-cleavage activity, which is restored with the addition of Telo G4. (e) Quantitative bar graph of recovery rates from figure d. Error bars are standard deviation (SD) (n = 3).

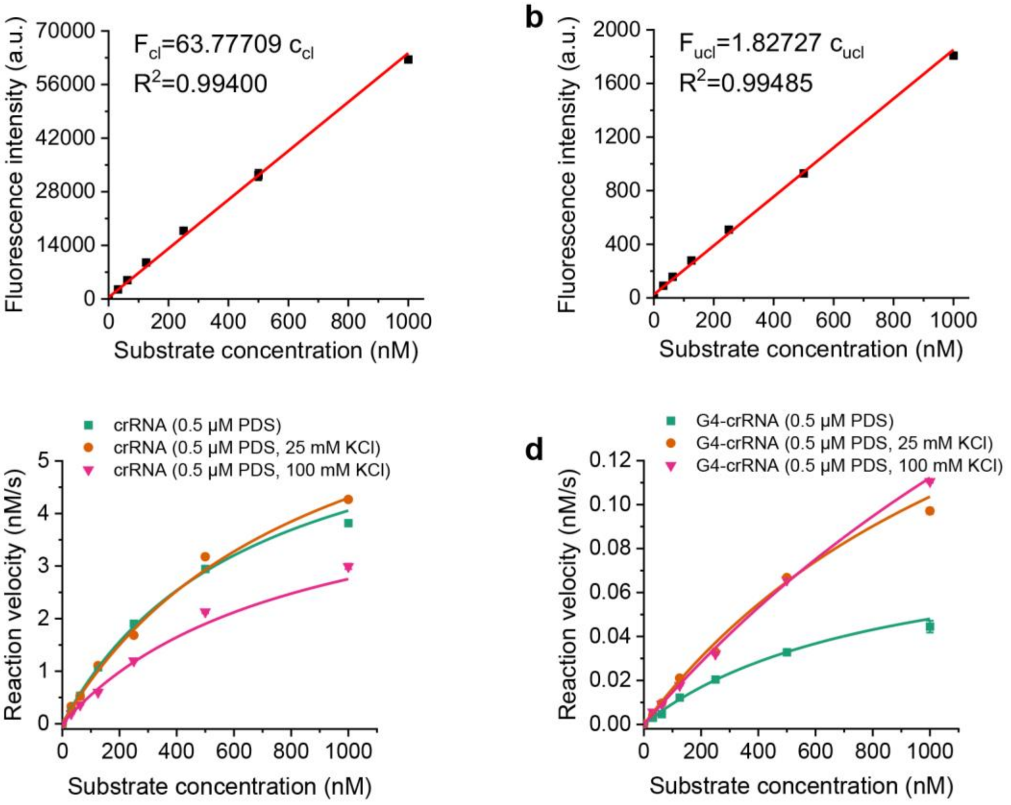

e

LbCas12a-crRNA / LbCas12a-G4-crRNA	k_{cat} (s^{-1})	K_M (M)	k_{cat}/K_M (M^{-1} s^{-1})	R^2
crRNA (0.5 μM PDS)	2.70 x 10^{-1}	0.66 x 10^{-6}	4.06 x 10^{5}	0.9963
G4-crRNA (0.5 μM PDS)	1.77 x 10^{-4}	0.85 x 10^{-6}	2.10 x 10^{2}	0.9966
crRNA (0.5 μM PDS, 25 mM KCl)	3.22 x 10^{-1}	0.87 x 10^{-6}	3.68 x 10^{5}	0.9930
G4-crRNA (0.5 μM PDS, 25 mM KCl)	5.12 x 10^{-4}	1.47 x 10^{-6}	3.48 x 10^{2}	0.9928
crRNA (0.5 μM PDS, 100 mM KCl)	2.00 x 10^{-1}	0.81 x 10^{-6}	2.46 x 10^{5}	0.9963
G4-crRNA (0.5 μM PDS, 100 mM KCl)	8.64 x 10^{-4}	2.85 x 10^{-6}	3.03 x 10^{2}	0.9984

Figure S6. (a) Background-subtracted fluorescence (F_{cl}) versus concentration of cleaved reporters (c_{cl}). (b) Background-subtracted fluorescence (F_{ucl}) versus concentration of uncleaved reporters (c_{ucl}). (c) Michaelis-Menten kinetics of LbCas12a *trans*-cleavage, aided by classical crRNA, under conditions of 0.5 μM PDS and 0, 25, or 100 mM KCl. (d) Michaelis-Menten kinetics of LbCas12a *trans*-cleavage, aided by G4-crRNA, under conditions of 0.5 μM PDS and 0, 25, or 100 mM KCl. Three technical replicates (n = 3) were measured for each substrate concentration, and the error bars represent the mean ± standard deviation (s.d.). The fitted curves are represented by solid lines, and the fitted parameters are provided in each panel. (e) Comparison of parameters in Michaelis-Menten kinetics for LbCas12a with varying crRNA and conditions.

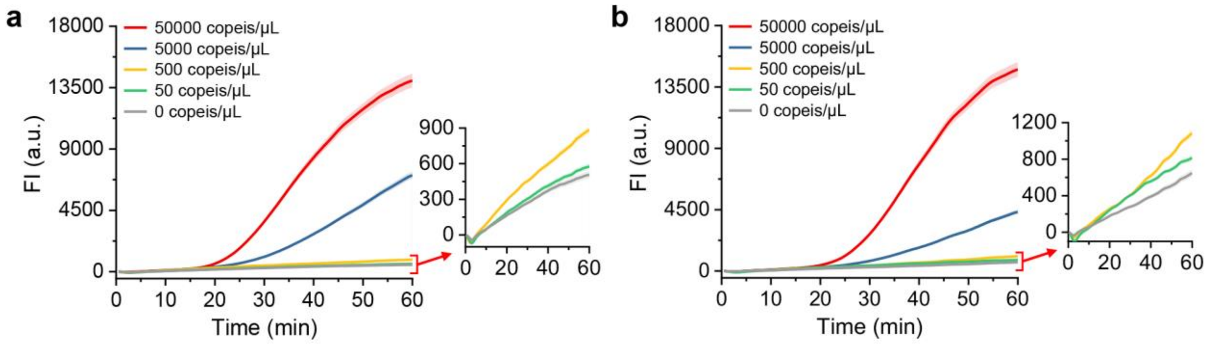

Figure S7. (a) Real-time fluorescence detection profiles of the asymmetric RPA-crRNA assisted CRISPR-Cas12a system, targeting the EBV target with different copy numbers, under the presence of PDS. (b) Real-time fluorescence detection profiles of the asymmetric RPA-G4-crRNA assisted CRISPR-Cas12a system, targeting the EBV target with different copy numbers, without PDS.

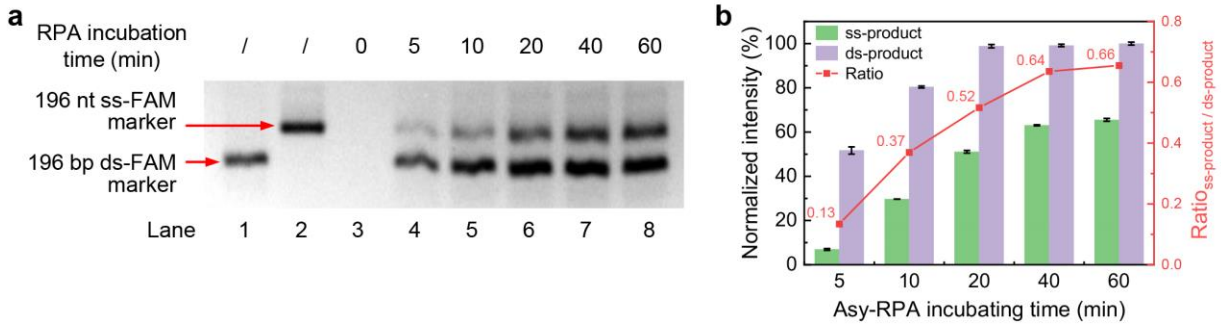

Figure S8. (a) Image of 3% agarose gel depicting the products of asymmetric RPA at various time points. Lane 1 contains a marker for 196 bp ds amplification products, while Lane 2 contains a marker for 196 nt ss amplification products. Lanes 3-8 represent the RPA reactions after 0, 5, 10, 20, 40, and 60 minutes, respectively. The quantities of both ss and ds amplification products vary over time, indicating the dynamics of RPA product formation. (b) Bar graph providing a precise quantitative analysis of the ss product yields from panel (a) at different reaction times. This data clearly demonstrates that our asymmetric RPA method successfully amplifies high concentrations of ss target sequences. Error bars are S.D. (n = 3).

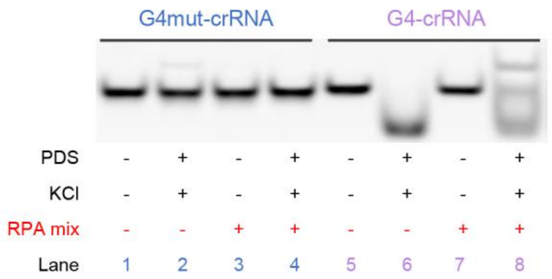

Figure S9. Gel imaging analysis validating the structures formed by G4mut-crRNA and G4-crRNA in the RPA mix, under conditions with and without PDS and potassium ions. Lane 1 and Lane 5 serve as control markers for G4mut-crRNA and G4-crRNA, respectively. Under the presence of PDS and potassium ions, G4-crRNA is capable of forming a stable RG4 structure (Lane 6), whereas G4mut-crRNA, which lacks G4 sequences, fails to form this structure (Lane 2). In the RPA mix (Lane 8), G4-crRNA also forms an RG4 structure in the presence of PDS and potassium ions, despite exhibiting minimal dissociation. This experiment confirms that the RG4 structure stabilized by PDS remains relatively stable in the RPA solution.

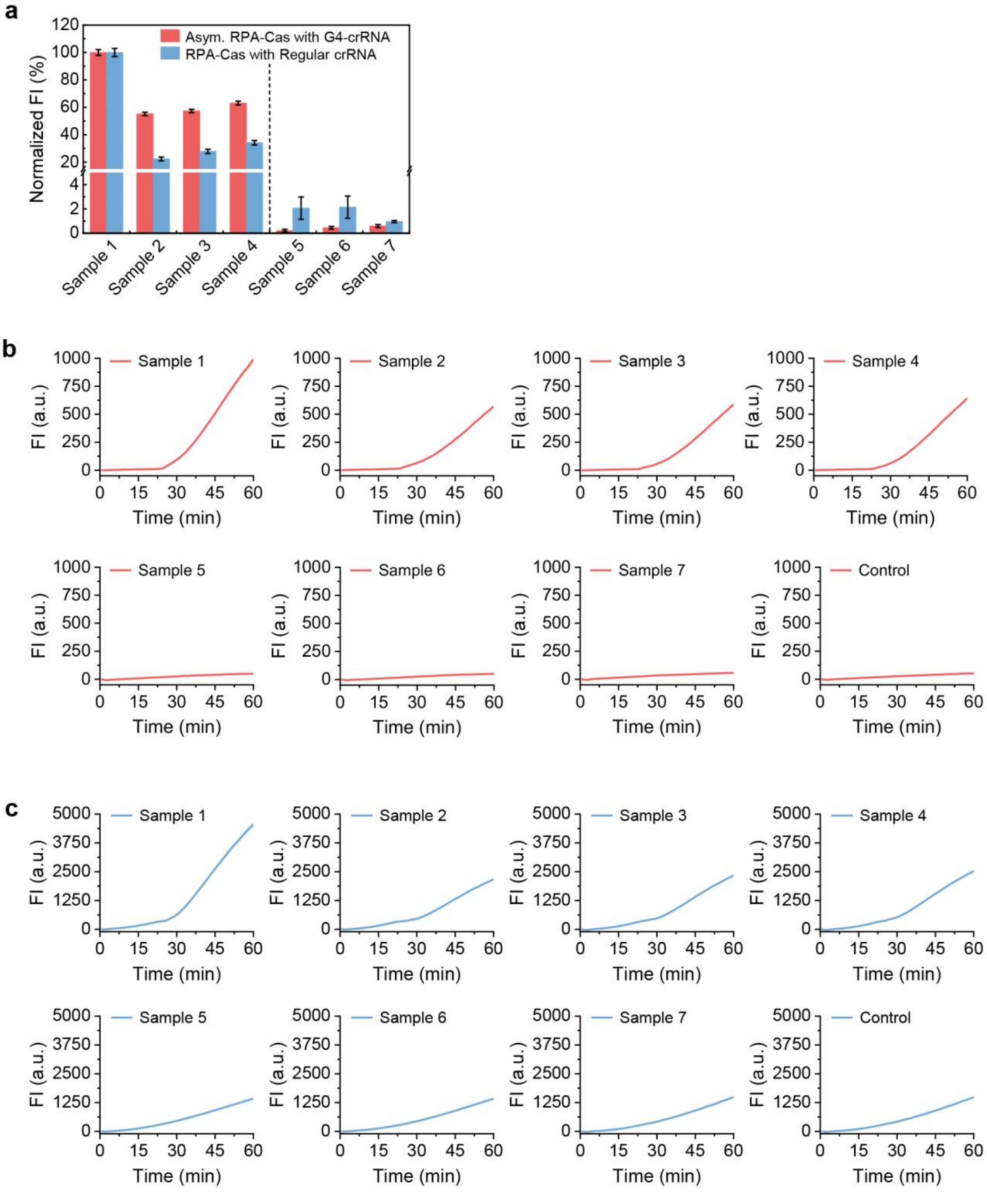

Figure S10. (a) Evaluation of our newly developed asymmetric RPA-Cas12a system using G4-crRNA (PDS+) alongside the traditional RPA-Cas12a system with crRNA on clinical samples. We collected plasma samples from seven patients, four of whom tested positive for EBV infection with high viral load

according to clinical qPCR results, indicated by the dashed line on the left side of panel (a). The remaining three patients were diagnosed as EBV negative, exhibiting low viral loads, as shown to the right of the dashed line. Our novel method aligns with both the traditional approach and clinical diagnostic outcomes. (b) Real-time fluorescence detection profiles for the asymmetric RPA-Cas12a system utilizing G4-crRNA (PDS+) targeting the seven clinical plasma samples. (c) Real-time fluorescence detection profiles for the classical RPA-Cas12a system with crRNA, also targeting the same seven clinical plasma samples. Error bars are S.D. (n = 3).

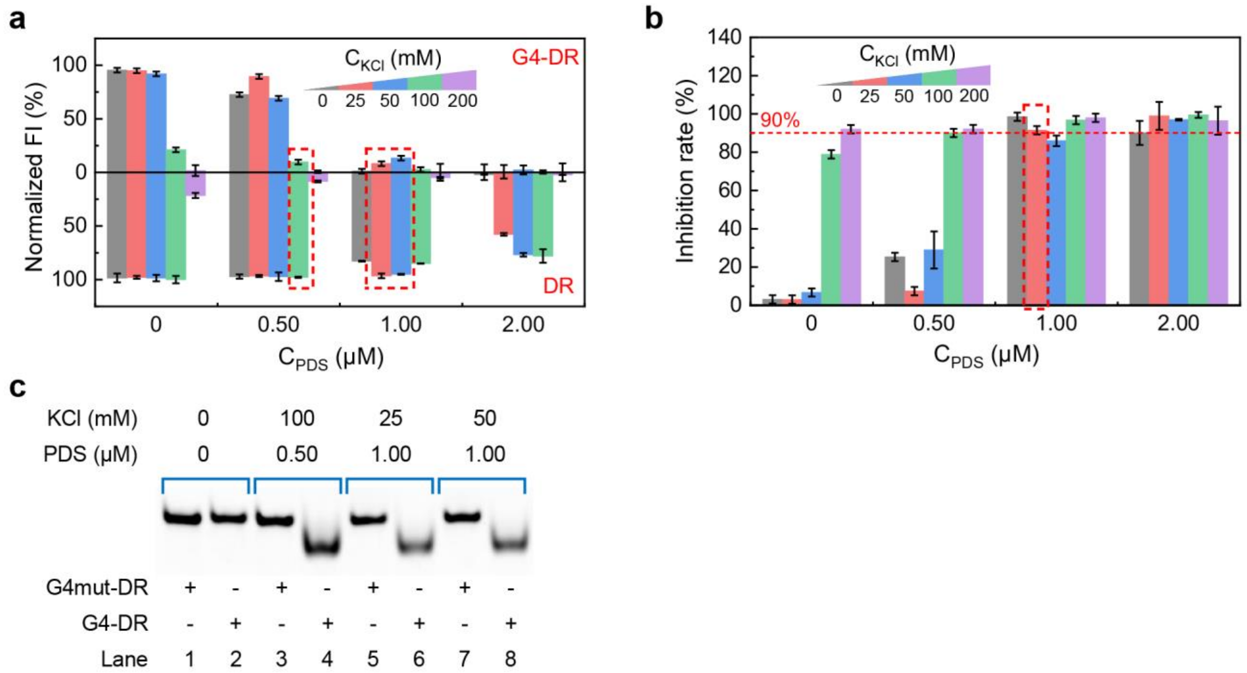

Figure S11. (a) Fluorescence bar chart comparing the cleavage efficiency of 5' RG4-containing G4-DR and classical DR on FQ (a single stranded DNA report) in response to varying concentrations of RG4 stabilizers such as PDS (0-2 μM), and KCl (0-200 mM) in the presence of the EBV spacer that targets the EBV target. Error bars are standard deviation (SD) (n = 3). (b) Inhibition rates of Cas12a *trans*-cleavage activity of G4-DR and classical DR under different concentrations of RG4 stabilizers—PDS (0-2 μM) and KCl (0-200 mM)—in the presence of the EBV spacer targeting the EBV target. Error bars are standard deviation (SD) (n = 3). (c) Native PAGE analysis of the secondary structures of G4-DR and its mutant variant G4mut-DR at PDS concentrations of 0-1 μM and KCl concentrations of 0-100 mM.

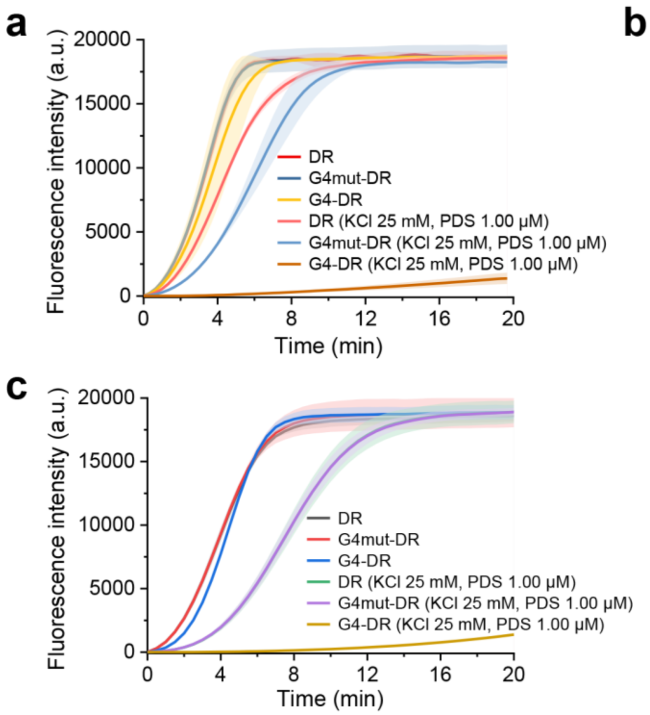

Figure S12. (a-c) Real-time fluorescence graphs illustrating the detection of respective target DNAs by G4-DR, G4mut-DR, and classical DR with their matched spacers for EBV (a), HCV (b), and MPXV (c), under conditions both with and without RG4 stabilizers. Error bars are standard deviation (SD) (n = 3).

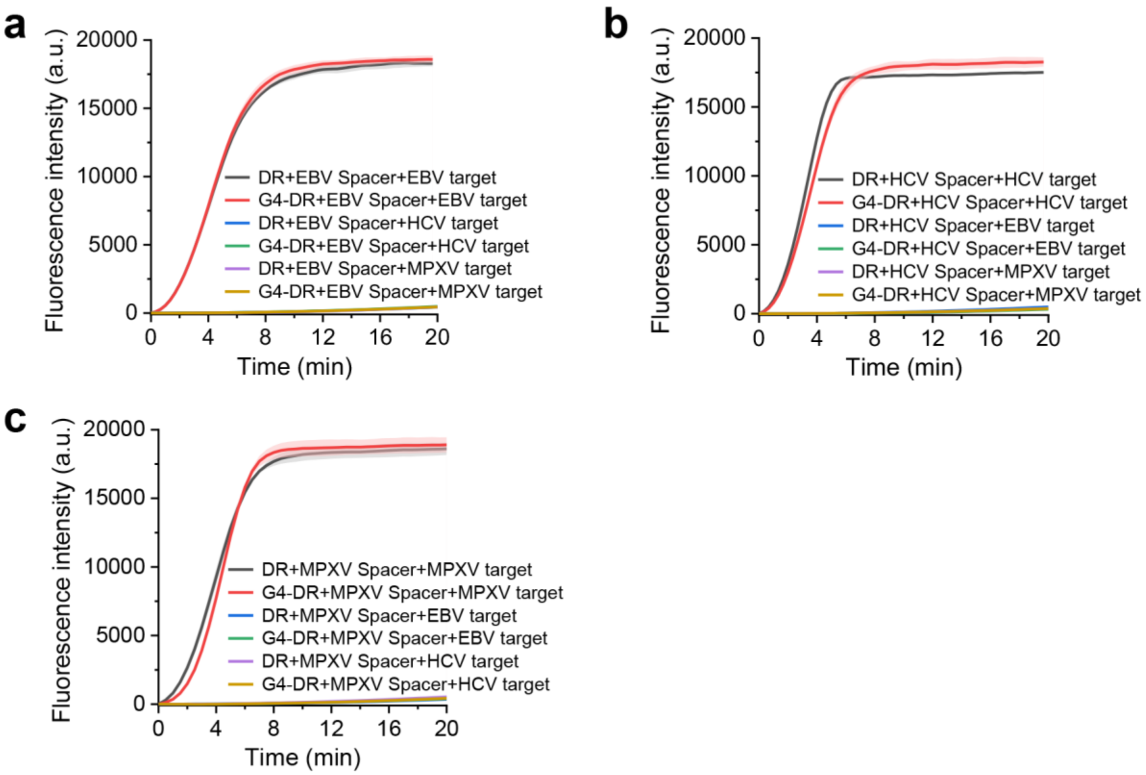

Figure S13. (a-c) Real-time fluorescence graphs depicting the detection of EBV, HCV, and MPXV targets by G4-DR and classical DR complexes matched with their respective spacers for EBV (a), HCV (b), and MPXV (c). Error bars are standard deviation (SD) (n = 3).

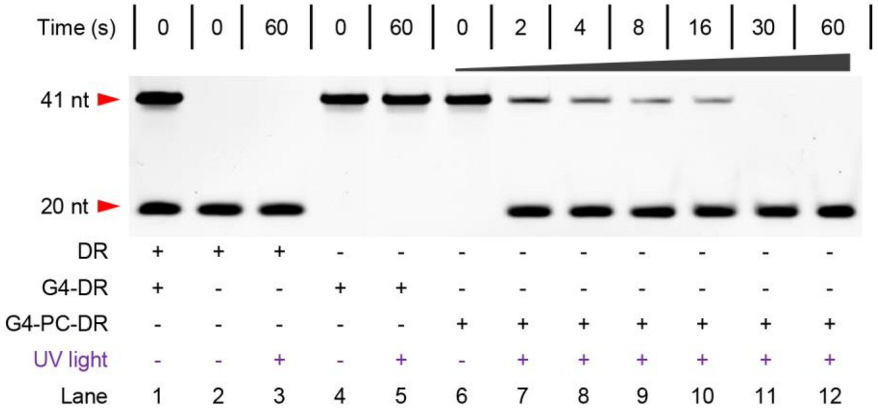

Figure S14. Denaturing PAGE analysis of the cleavage status of the PC linker in G4-PC-DR upon UV exposure (365 nm, 35W) for durations ranging from 0 to 60s.

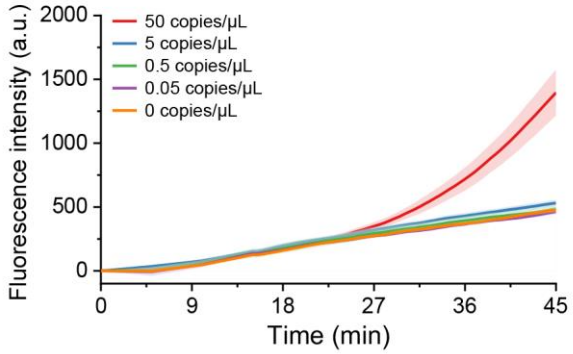

Figure S15. Real-time fluorescence images of the CRISPR-Cas12a system assisted by classical RPA-crRNA for the detection of EBV target. Error bars are standard deviation (SD) (n = 3).

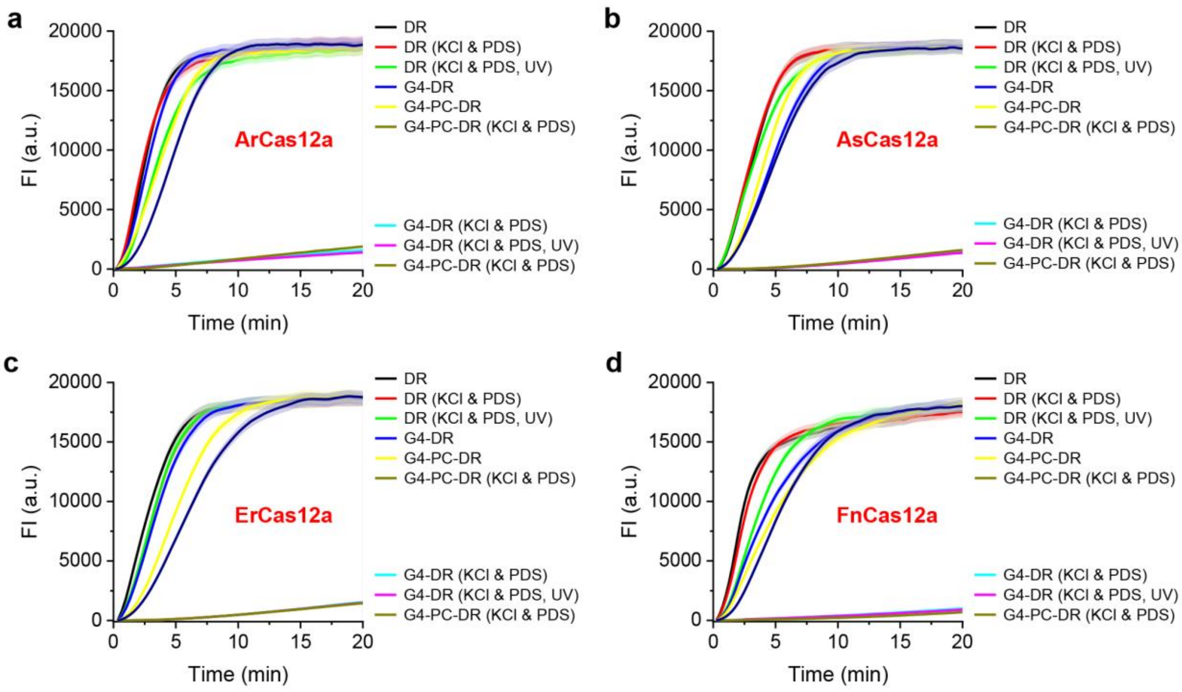

Figure S16. Real-time fluorescence signals of *trans*-cleavage activity by various Cas12a variants—ArCas12a (a), AsCas12a (b), ErCas12a (c) and FnCas12a (d)—when utilizing both classic split crRNA (DR+Spacer) and our newly developed G4-PC-DR+Spacer, under conditions with and without PDS and potassium ions, as well as under UV light exposure or in the absence of light. The results illustrate that G4-PC-DR shows a level of inhibition comparable to LbCas12a across all tested Cas12a proteins in the presence of PDS and potassium ions. Notably, treatment with UV light restored enzymatic activity following inhibition. Although there are slight variations in fluorescence readings among the different Cas12a subtypes, our findings indicate that this approach is likely applicable to other Cas12a variants as well.

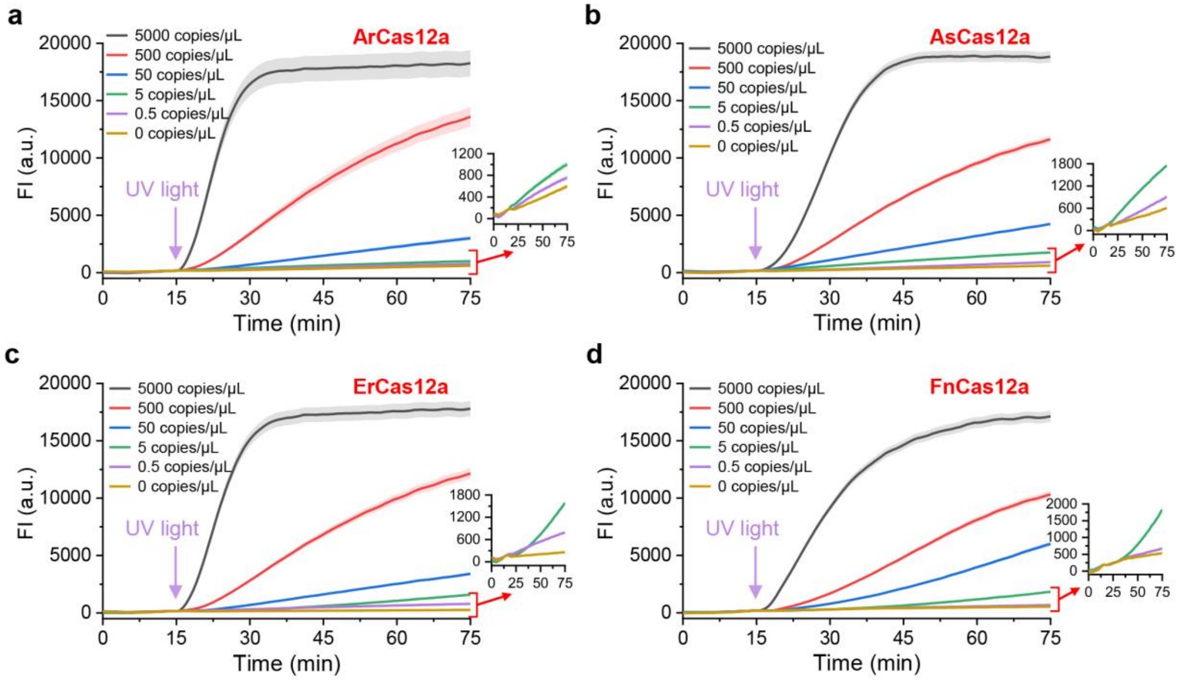

Figure S17. Real-time fluorescence signals obtained from the one-pot RPA-CRISPR nucleic acid detection method utilizing G4-PC-DR (PDS+) and various Cas12a variants—ArCas12a (a), AsCas12a (b), ErCas12a (c), and FnCas12a (d)—for the detection of different concentrations of EBV standards. The reaction involved a 15-minute RPA followed by a 30-second exposure to UV light and continued incubation at 37°C. All variants successfully detected as low as 0.5 copies/μL.

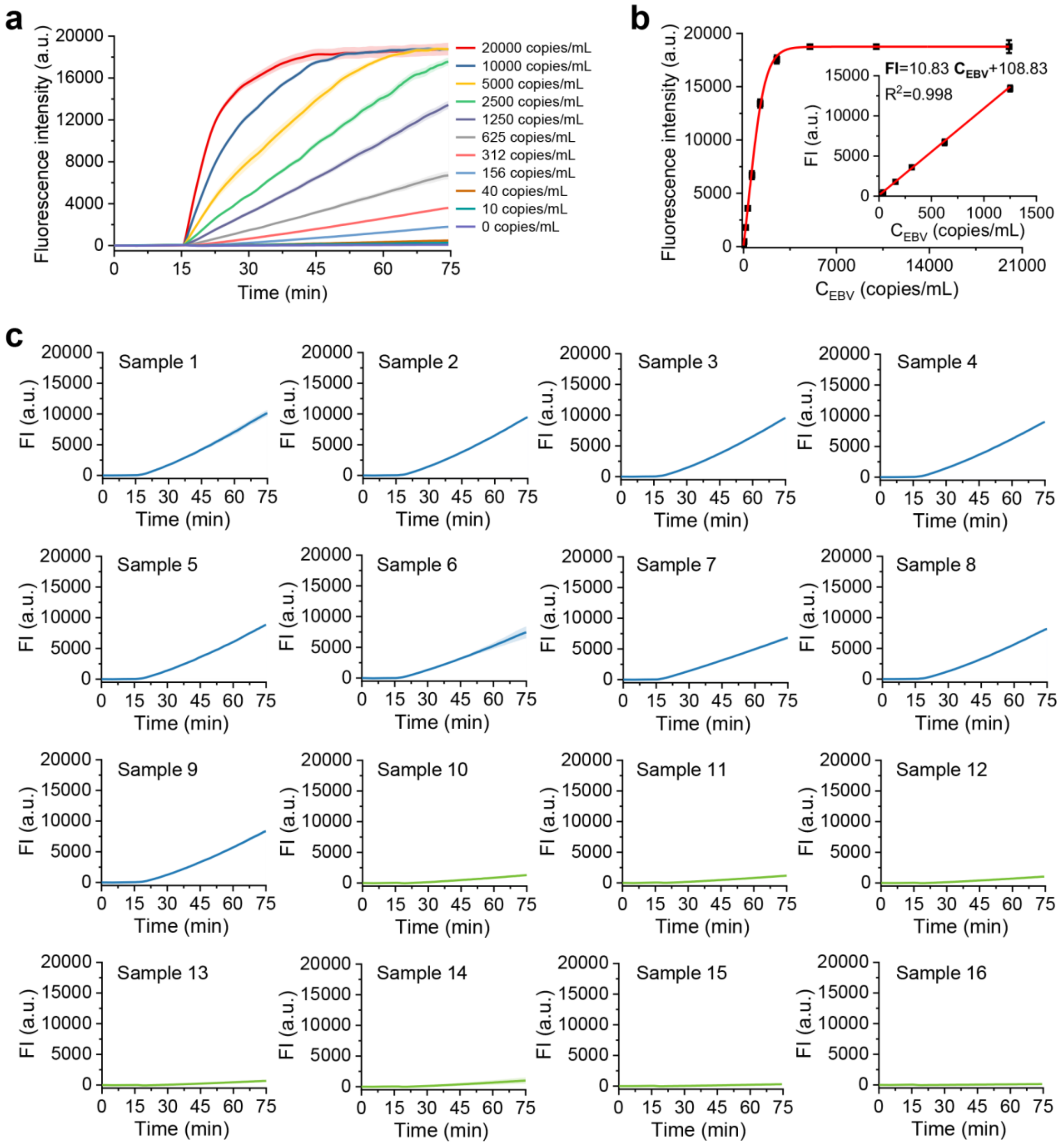

Figure S18. (a) Real-time fluorescence images of the CRISPR-Cas12a system assisted by RPA-G4-PC-DR for the detection of EBV target standard samples with varying copy numbers. (b) Graph showing the linear relationship between fluorescence values and EBV target concentration in (a). (c) Original fluorescence data records of the detection of EBV concentration in 9 EBV-infected patient samples and 7 healthy donors' blood samples using the RPA-G4-PC-DR assisted CRISPR-Cas12a system. Error bars are standard deviation (SD) (n = 3).

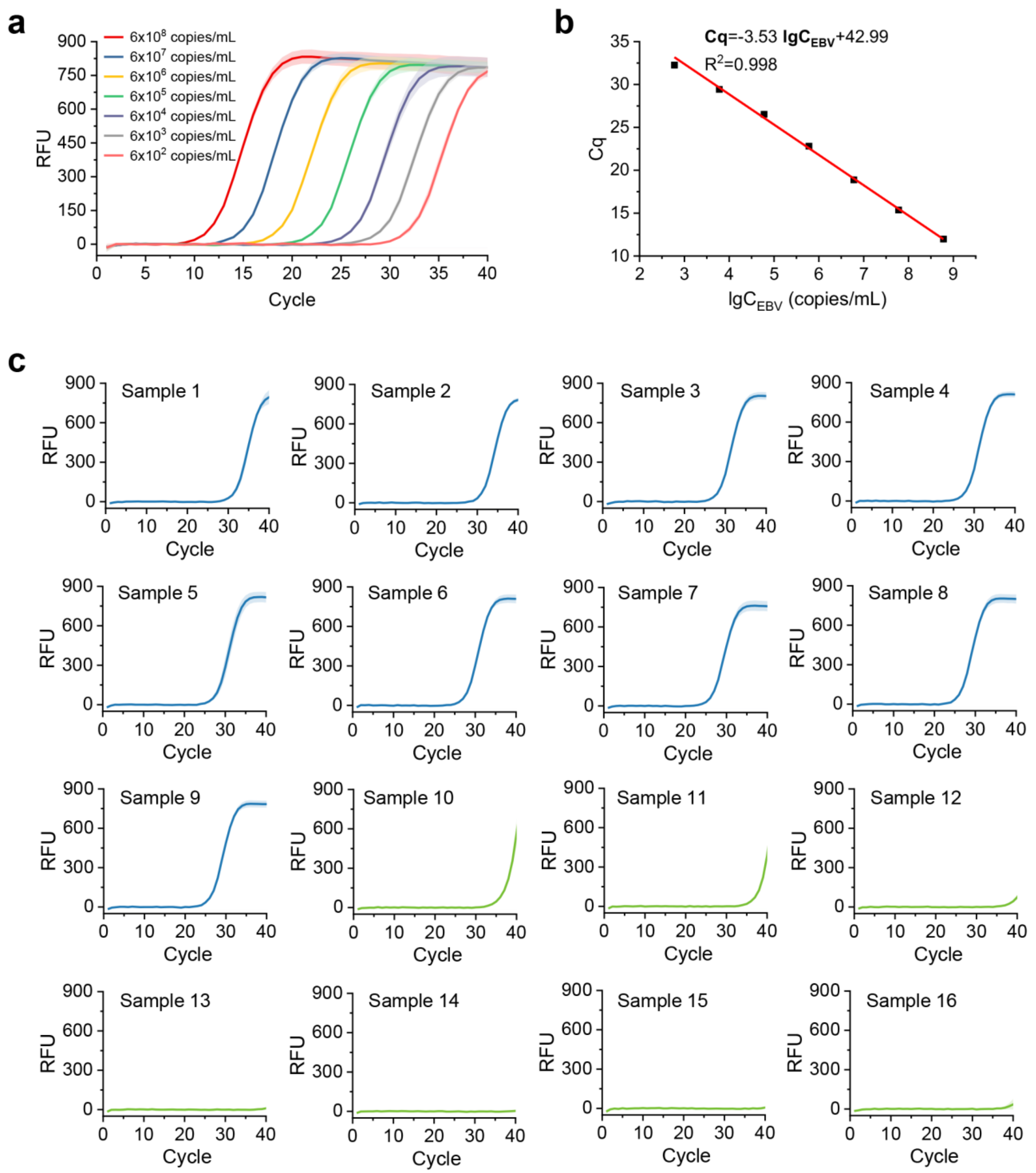

Figure S19. (a) Real-time fluorescence images of the classical qPCR method for the detection of EBV target standard samples with varying copy numbers. (b) Graph showing the linear relationship between fluorescence values and EBV target concentration from (a). (c) Original fluorescence data records of the detection of EBV concentration in 9 EBV-infected patient samples and 7 healthy donors' blood samples using the classical qPCR method. Error bars are standard deviation (SD) (n = 3).

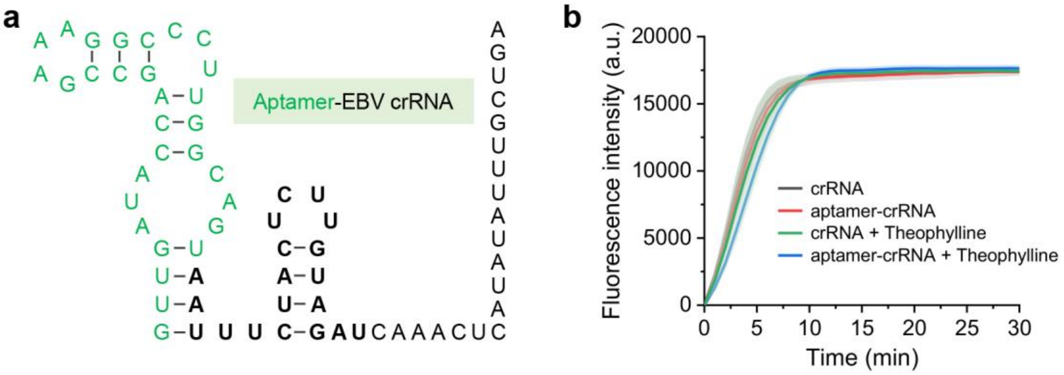

Figure S20. (a) Schematic representation of the crRNA incorporating a theophylline RNA aptamer at its 5' end for targeting EBV. The green region denotes the theophylline RNA aptamer, the bold black section indicates the DR, and the remaining black section represents the spacer targeting EBV. (b) Real-time fluorescence signals exhibit the performance of theophylline RNA aptamer-crRNA compared to traditional crRNA, under both the presence and absence of theophylline during the recognition of the EBV target. The data indicate that the incorporation of the theophylline RNA aptamer at the 5' end of crRNA does not impact Cas12a activity, regardless of theophylline presence.

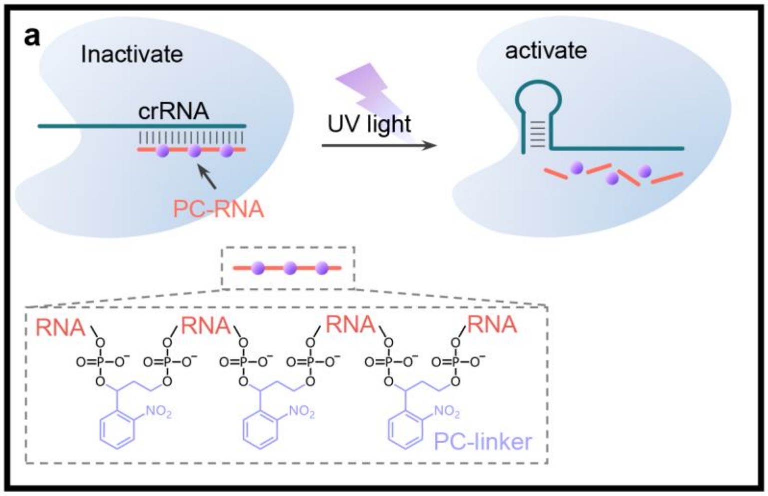

(a) Method: RPA-CRISPR
Characteristics:
1. Introduction of PC linker-modified RNA.
2. Achieves temporary inactivation of Cas protein in the RPA-CRISPR one-pot method.
3. Inactivation is reversed by UV light, activating the CRISPR reaction.
4. First-time implementation of this technique.
Reference:
Hu et al, *Proc. Natl. Acad. Sci. U. S. A.* **2022**, 119 (26), e2202034119.

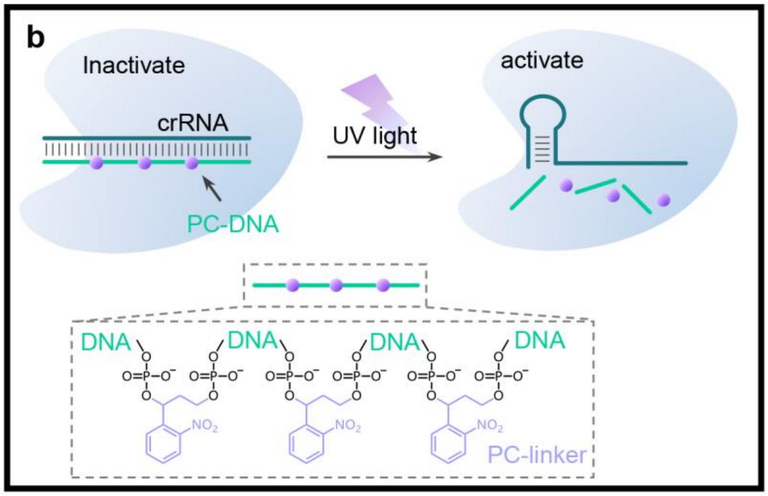

(b1) Method: RPA-CRISPR
Characteristics:
1. Utilizes PC linker-modified DNA that complements full-length crRNA.
2. DNA modification reduces overall costs compared to RNA.
Reference:
Chen et, *Anal. Chem*. **2022**, 94 (27), 9724-9731.
(b2) Method: RCA-CRISPR
Characteristics:
1. Employs PC linker-modified DNA matching full-length crRNA.
2. Introduces a rolling circle amplification (RCA) approach instead of RPA.
3. Maintains lower costs through complementary DNA use.
Reference:
Wu et al, *Chem. Eng. J.* **2023**, 477, 146814

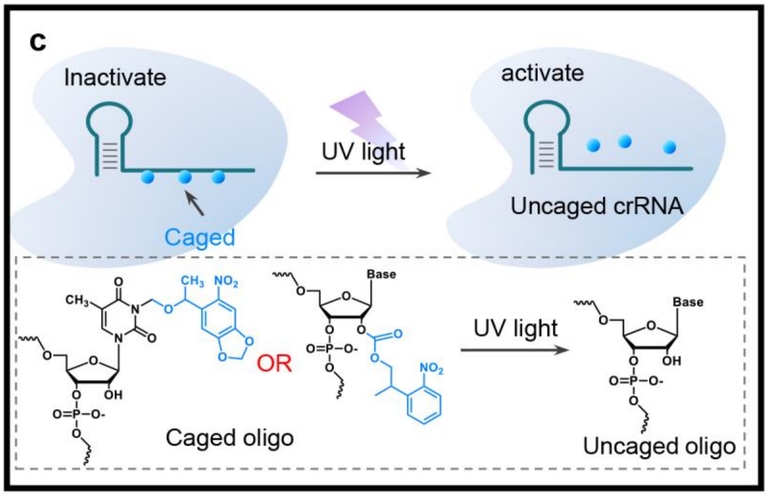

(c1) Method: RPA-CRISPR
Characteristics:
1. No additional nucleic acids needed.
2. Temporarily inhibits Cas protein activity by replacing Us with photosensitive artificial Ts in crRNA.
3. UV light exposure activates the CRISPR reaction post-RPA amplification.
4. Limitation: Requires original Spacer region to contain U.
Reference:
Hu et al, *Angew. Chem. Int. Ed.* **2023**, 62 (23), e202300663.
(c2) Method: RPA-CRISPR
Characteristics:
1. Eliminates necessity for additional nucleic acids or base substitutions.
2. Reacts normal crRNA with developed reagent to yield caged RNA.
3. Effective temporary inhibition of Cas activity, activated by UV exposure.
4. Overcomes prior limitations; still necessitates initial treatment for varying crRNAs.
Reference:
Liu et al, *Angew. Chem. Int. Ed.* **2024**, e202401486.

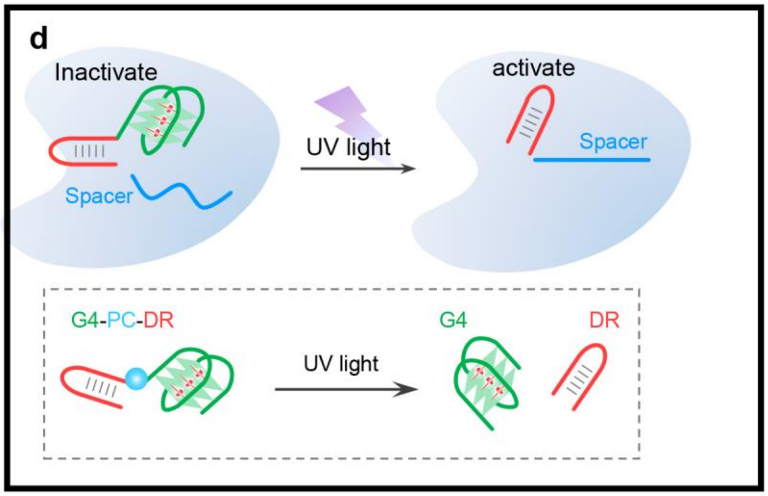

(d) Method: RPA-CRISPR
Characteristics:
1. Does not require additional nucleic acids, base substitutions, or labeling reactions.
2. Uses a single commercially available G4-PC-DR preparation to simplify the process.
3. Allows for low-cost acquisition of split 20-22 nt Spacer region for different target detection.
4. Reduces preparation costs and operational complexity, enhancing system universality.
Reference:
This work.

Figure S21. A comparative summary of UV-controlled isothermal amplification methods for CRISPR-Cas12a one-pot nucleic acid diagnostics developed in recent years.
